# MICAL2 is essential for myogenic lineage commitment

**DOI:** 10.1038/s41419-020-02886-z

**Published:** 2020-08-18

**Authors:** Nefele Giarratana, Filippo Conti, Rita La Rovere, Rik Gijsbers, Paolo Carai, Robin Duelen, Tim Vervliet, Geert Bultynck, Flavio Ronzoni, Roberto Piciotti, Domiziana Costamagna, Stefania Fulle, Ivana Barravecchia, Debora Angeloni, Yvan Torrente, Maurilio Sampaolesi

**Affiliations:** 1 Translational Cardiomyology, Department of Development and Regeneration, KU Leuven, Leuven, Belgium; 2 Stem Cell Laboratory, Department of Pathophysiology and Transplantation, University of Milan, Unit of Neurology, Fondazione IRCCS Ca’ Granda Ospedale Maggiore Policlinico, Centro Dino Ferrari, Milan, Italy; 3 Department of Neuroscience, Imaging and Clinical Sciences, Interuniversity Institute of Miology (IIM), University “G d’Annunzio”, Chieti, Italy; 4 Human Anatomy Unit, Department of Public Health, Experimental and Forensic Medicine, University of Pavia, Pavia, Italy; 5 Laboratory of Molecular and Cellular Signaling, Department of Cellular and Molecular Medicine, and Leuven Kanker Instituut, KU Leuven, 3000 Belgium; 6 Laboratory for Molecular Virology and Gene therapy, Department of Pharmaceutical and Pharmacological Sciences, KU Leuven, Leuven, Belgium; 7 Department of Cardiovascular Sciences, KU Leuven, 3000 Leuven, Belgium; 8 Department of Biomedical Sciences, Humanitas University, Rozzano, Italy; 9 MedLab, Institute of Life Sciences, Scuola Superiore Sant’Anna, Pisa, Italy

**Keywords:** Mechanisms of disease, Molecular biology

## Abstract

Contractile myofiber units are mainly composed of thick myosin and thin actin (F-actin) filaments. F-Actin interacts with Microtubule Associated Monooxygenase, Calponin And LIM Domain Containing 2 (MICAL2). Indeed, MICAL2 modifies actin subunits and promotes actin filament turnover by severing them and preventing repolymerization. In this study, we found that MICAL2 increases during myogenic differentiation of adult and pluripotent stem cells (PSCs) towards skeletal, smooth and cardiac muscle cells and localizes in the nucleus of acute and chronic regenerating muscle fibers. In vivo delivery of Cas9–Mical2 guide RNA complexes results in muscle actin defects and demonstrates that MICAL2 is essential for skeletal muscle homeostasis and functionality. Conversely, MICAL2 upregulation shows a positive impact on skeletal and cardiac muscle commitments. Taken together these data demonstrate that modulations of MICAL2 have an impact on muscle filament dynamics and its fine-tuned balance is essential for the regeneration of muscle tissues.

## Introduction

Muscle tissue represents 40% of human body mass and provides locomotion, posture support and it is required for breathing. Myogenesis is the process that ensures the generation of myoblasts which differentiate into skeletal muscle tissue^1^. Once the muscle is mature, a sub-population of *Pax7*^+^ cells becomes quiescent as satellite cells (SCs) and muscle renewal and regeneration rely on the activated SCs that have the potential to fuse and form new fibers, as well as maintaining the stem cell niche^2–4^. This process occurs in adult tissue when muscles are damaged^5^ to restore the damaged contractile myofiber units^6^. Thin actin filaments (F-actin) are crucial components of contractile myofiber units. F-Actin interacts with Microtubule Associated Monooxygenase, Calponin And LIM Domain Containing proteins (MICALs), capable to make oxidation-reduction reactions (redox) by its FAD domain^7,8^. MICALs make redox reactions on filamentous-actin (F-actin), by binding FAD and using NADPH and O_2_ to depolymerize F-actin^9–12^. MICALs were seen involved in many functions within different cell types, all depending on dynamic actin cytoskeleton remodeling^8,11–16^. So far, MICAL was identified only in Drosophila skeletal muscles^14,17^ and its role in mammalian muscles is totally unexplored. However, it seems that MICALs are indirectly involved in cardiovascular integrity by regulating semaphorin 3a expression. Indeed, semaphorin 3a overexpression led to a reduction of post-myocardial infarction arrhythmia^18,19^. Nevertheless, evidences of a direct role of MICALs on cardiac and smooth muscle are still lacking. Among the MICALs, MICAL2 emerged as the one involved in angiogenesis, cell viability, gene transcription^19^. Indeed, MICAL2 modifies actin subunits and promotes actin turnover by ensuring disaggregation and preventing repolymerization^11^. In addition, in a genome-wide profiling study, MICAL2 was found upregulated among a set of ten functionally linked genes involved in the disease evolution of mdx-mice, a mouse model of Duchenne muscular dystrophy (DMD)^20^.

We hypothesize here that the striking capability of MICAL2 to connect oxygen availability with F-actin depolymerization, and hence cytoskeleton dynamics, was implicated into the process of myogenic differentiation. Therefore, we speculated that MICAL2 was involved in smooth, skeletal and cardiac muscle differentiations. Moreover, gaining this knowledge might help to understand the role of MICAL2 in muscle pathological conditions, including muscular dystrophies (MDs).

We characterized firstly the presence of MICAL2 during differentiation towards skeletal, smooth and cardiac muscle cells from myogenic progenitors. Secondly, we assessed MICAL2 expression in dystrophic conditions where the pool of adult stem cells was exhausted. We then proceeded further with MICAL2 depletion and overexpression studies and employed H11^LSL-Cas9^ mice for the delivery of Cas9–Mical2 guide RNA complexes. Our data point to MICAL2 as a regulator of myogenic differentiation, also outlining its multifaceted effects in determining the cellular response to the environment. In particular, in the pathophysiological context MICAL2 affects proliferation and cell migration, and controls muscle regeneration.

## Materials and methods

### Cell isolation, culture and differentiation

Murine satellite cells (mSCs) were isolated after muscle double enzymatic digestion of TA, GC and Q of 4–6-week-old male C57BL/6 mice. Digestions of the minced muscles were done with 0.02% collagenase D (Sigma Aldrich) and 0.06% of pancreatin (Sigma Aldrich) dissolved in sterile PBS, for 1 h and for 30 min, respectively. Between first and second digestion a filtration with a 70 µm strainer was done. Cells were grown in high glucose Dulbecco’s Modified Eagle’s Medium (D-MEM) supplemented with 20% FBS, 1% penicillin/streptomycin solution [100 units], 2 mmol/L glutamine, 1 mmol/L sodium pyruvate and 1% chicken embryo extract (all from Thermo Fisher Scientific, except otherwise specified).

Murine female C2C12 skeletal myoblasts (ATCC, Manassas, VA, USA) were grown in high glucose DMEM supplemented with 10% FBS, 1% pen/strep, 1% sodium pyruvate, 2 mM l glutamine. The two cell types were maintained at 37 °C in a humidified atmosphere of 5% CO_2_. Differentiation to myotubes was induced by shifting 80% confluent cultures to DMEM supplemented with 2% horse serum. The medium was changed every other day, and within 2 and 5 days—for mESCs and C2C12 cells, respectively—most of the cells were fused into myotubes.

Murine male MABs were previously isolated in the translational cardiomyology laboratory^21^. We cultured and expanded these cells on collagen-coated plates in high glucose DMEM culture medium supplemented with 20% FBS, 1% penicillin/streptomycin solution [100 units], 2 mmol/L glutamine, 1 mmol/L sodium pyruvate, 1% NEAA, 0.5% β-mercaptoethanol. When cells reached 80% to 85% confluence, they were split in a 1:4 ratio. To induce smooth muscle differentiation, we plated 5 × 10^4^ cells/ cm^2^ in collagen-coated plates, incubated at 37 °C with maintenance medium. After 24 h, cells underwent media change with smooth muscle differentiation medium for 8 days. Smooth-muscle differentiation medium consists of high glucose DMEM, supplemented with 2% horse serum, 1% penicillin/streptomycin solution, 2 mmol/l glutamine, 1 mmol/L sodium pyruvate, and 10 ng/ml TGF-b (Peprotech, Oak Park, California). Medium change was every day.

Murine ESCs were derived from blastocyst of C57/Bl6 embryos following the methods published by Kohueiry et al.^22^ and kindly given to our use by professor Koh’s laboratory—Stem Cell Institute KU Leuven (SCIL), Leuven, Belgium. mESCs were maintained with feeder cells in Glasgow’s Modified Eagle’s Medium (G-MEM) supplemented with 7.5% FBS, 1% penicillin/streptomycin solution [100 units], 2 mmol/L glutamine, 1 mmol/L sodium pyruvate, 1% NEAA, 1:500 β-mercaptoethanol and 1:10000 Leukemia inhibitory factor (LIF) was freshly added to every media change. Maintenance media change was every day. Prior to cardiac differentiation, 2 days adaptation on feeder-free gelatin-coated wells in serum maintenance media was done. Cardiac differentiation was induced by embryoid bodies (EBs) formation via hanging drop method, putting in one drop (20 µl/ drop) ~500 cells. Each drop was forming one EB. EBs were grown in cardiac differentiation medium for 48 h in hanging drops. Drops were then collected in cardiac differentiation medium the second day, to be grown in suspension in ultra-low attachment (ULA) plates for 5 days. Cardiac differentiation medium consists of G-MEM supplemented with 20% FBS, 1% penicillin/streptomycin solution [100 units], 2 mmol/L glutamine, 1 mmol/L sodium pyruvate, 1% NEAA and 1:500 β-mercaptoethanol. Medium change was every 2 days. At day 5 of ULA plate culture, EBs were culture in adhesion in gelatin-coated plates. From day 9, the first beating areas appeared. Cells were kept in culture until day 11 of cardiac differentiation.

### Quantitative real-time PCR

For quantitative Reverse Transcription Polymerase Chain Reaction (qRT-PCR) assays, RNA was isolated through Purelink^®^ RNA mini kit (Thermo Fisher Scientific), and treated with Turbo™ DNA-free kit (Thermo Fisher Scientific) to purify RNA samples. 1 μg RNA was reverse-transcribed using Superscript III Reverse Transcriptase First-Strand Synthesis SuperMix (Thermo Fisher Scientific). After having cDNA from the reverse transcription, qRT-PCR was performed with the Platinum SYBR Green QPCR SuperMix-UDG (Thermo Fisher Scientific). The oligonucleotide primer sequences are listed in Table S1. Relative expression values were obtained by normalizing Ct values of the tested genes to Ct values of the housekeeping genes *Gapdh, Hprt and Tbp*.

### Immunofluorescence assay

For both in vitro cultures and tissue cryosections, cells were fixed with 4% paraformaldehyde for 15 min at room temperature nd after three PBS washes, permeabilization by 1% Bovine Serum Albumin (BSA) + 0.2% or 0.5% triton was done for 30–45 min at RT to increase permeability. Cells were then blocked for 30 min with 10% donkey serum at room temperature followed by overnight incubation at 4 °C with different primary antibodies at the indicated dilution listed in Table S2. Secondary antibody incubation was done by using the appropriate Alexa Fluor 488-, 594- and/or 647-conjugated secondary antibody (Thermo Fisher Scientific; 4 μg/mL). Nuclei were stained with Hoechst (33342, Thermo Fisher Scientific; 1:10000) for 1 min. Analyses were assessed using an Eclipse Ti Microscope and NIS-Elements AR 4.11 Software (both from Nikon). Confocal images were obtained by using ZEISS LSM 800 with Airyscan microscope. Images were quantified and analyzed using ImageJ software.

### Western blotting

Western blotting (WB) analyses were performed on cell or tissue lysates using RIPA buffer (Sigma-Aldrich) supplemented with 10 mM Sodium Fluoride, 0.5 mM Sodium Orthovanadate, 1:100 Protease Inhibitor Cocktail, and 1 mM phenylmethylsulfonyl fluoride (PMSF). The same amount of protein samples (40 µg) have been heat-denatured in sample-loading buffer (50 mM Tris–HCl, pH 6.8, 100 mM DTT, 2% SDS, 0.1% bromophenol blue, 10% glycerol). SDS-polyacrylamide gel electrophoresis was used for resolving and then proteins were transferred to nitrocellulose membranes (Protran, Nitrocellulose membrane, Sigma-Aldrich). Membranes were blocked with Tris-buffered saline (TBS) containing 0.05% Tween and 5% skim milk powder (Sigma-Aldrich). This step has been followed by overnight incubation with different primary antibodies at the indicated dilution listed in Table S2 (see also key resources table). All secondary horseradish peroxidase (HRP)-conjugated antibodies (BioRad) were diluted 1:5000 in TBS-Tween and 2.5% skim milk powder. After incubation with SuperSignal Dura Chemiluminescence substrate (Thermo Scientific), the polypeptide bands were detected with GelDoc chemiluminescence detection system (BioRad). Quantitation was performed on gels loaded and blotted in parallel. Relative densitometry was obtained by normalizing the protein band versus background and the housekeeping protein α-TUBULIN using the QuantityOne software (BioRad).

### Histological and immunofluorescence analyses

All animal procedures were performed at the Translational Cardiomyology laboratory according to the guidelines of the Animal Welfare Committee of KU Leuven (Ethical Committee approval number P161/2018.) and Belgian/European legislation. Mice were sacrificed by cervical dislocation, skeletal muscles and hearts were snap frozen in liquid nitrogen-cooled isopentane and kept at −80 °C for further analyses. The samples were cut transversally in 7 µm sections using a cryostat machine (Leica, Wetzlar, Germany). Immunofluorescence analyses were performed to characterize MICAL2 in skeletal muscle of different murine models and to check MICAL2 protein in AAV-Mical2 H11^Cas9^ and in AAV-SHAM H11^Cas9^ overtime, not only in skeletal muscle, but also in hearts. Moreover, cryosections were also used for Hematoxylin & Eosin staining and Picro-Sirius red staining on AAV-Mical2 H11^Cas9^ and in AAV-SHAM H11^Cas9^ skeletal and cardiac muscles.

### Design, cloning and production of transfer plasmids for rAAV production pAAV-TF_U6-Mical sgRNA2 exon 3-TnnT4-intron-3flag-Cre and pAAV-TF_U6-SHAM-sgRNA TnnT4-intron-3flag-Cre

All enzymes were purchased from (Thermo Fisher Scientific; Brussels; Belgium). All viral vectors were designed and produced by the Leuven Viral Vector Core. The integrity of all plasmids was verified by DNA sequencing.

We first generated a universal AAV-transfer plasmid to clone gRNAs driven by a U6 promoter, combined with a Cre recombinase driven from a specific promoter (TnT4; https://academic.oup.com/cardiovascres/article/104/1/15/317207) together with a mCherry reporter, pAAV-TF-U6-gRNA-shuttle-TnT4-intron-mCherry-NLSx3-myc-IRES-Cre. To do so, we amplified of the U6-gRNA region from plenti-CrispR-v2 (Addgene plasmid #52961) by PCR using NheI-U6-gRNA-shuttle primer sense (101-057_NheI-U6-s: aaaaaaGCTAGC tttcccatgattccttcatatttgc) and NheI-U6-gRNA-shuttle primer asense (101-058_U6-EcoRI-NheI-as: aaaaaagctagcgaattcaaaaaagcaccga). The resulting amplicon was digested by NheI and cloned upstream of the TnT4 promoter in pAAV-TF-TnT4-intron-mCherry-NLSx3-myc-IRES-Cre (also NheI digested), generating pAAV-TF-U6-gRNA-shuttle-TnT4-intron-mCherry-NLSx3-myc-IRES-Cre.

Next, the cDNA cassette (mCherry-NLSx3-myc-IRES-Cre) was digested using XbaI and MluI, and replaced by 3xflag-Cre cDNA. In a first step, we amplified and cloned Cre cDNA (lacking an ATG) using XbaI-Cre-s (aaaaaaTCTAGAgaattcCGTACGcccaagaagaa gaggaaggtgtcc) and Cre-MluI-as (aaaaaacgcgtactagttcagtcaccatcttcgagc), and next reintroduced the ATG together with the 3xflag tag using oligo’s (XbaI-3xflag-Pfl23II oligo: Ctagaatggactacaaagaccatgacggtgattataaagatcatgatatcgattacaaggatgacgatgacaagc and XbaI 3xflag-Pfl23II oligo: gtacgcttgtcatcgtcatccttgtaatcgatatcatgatctttataatcaccgtcatggtctttgtagtcc att). Together this cloning resulted in pAAV-TF-U6-gRNA-shuttle-TnT4-intron-3xflag-Cre. Specific gRNAs were cloned as adaptors following oligo ligation to be driven by U6 using Esp3I digestion of pAAV-TF-U6-gRNA-shuttle-TnT4-intron-3xflag-Cre. One the one hand, Mical2 sgRNA2_exon 3, and as a negative/sham control a sgRNA directed against eGFP (https://www.addgene.org/crispr/reference/grna-sequence/), resulting in two transfer plasmids for AAV-vector production, pAAV-TF_U6-Mical sgRNA2 exon 3-TnT4-intron-3flag-Cre and pAAV-TF_U6-SHAM-sgRNA TnT4-intron-3flag-Cre.

Recombinant rAAV production was performed using standard procedures. Briefly, HEK 293 T cells (ATCC, Manassas,VA, USA) were seeded at 1e08 cells in a Hyperflask Cell Culture Vessel (1720 cm2, Corning Life Sciences, Kennebunk, ME, USA) and transfected with AAV-TF, AAVrep/cap and pAdbDeltaF6 plasmids in a 1:1:1 ratio using Optimem (Invitrogen, Merelbeke, Belgium). After 24 h of incubation, the medium was replaced. The supernatant was harvested 3 days after transient transfection and concentrated by tangential flow filtration. The concentrated supernatant was next purified using an iodixanol step gradient. The gradient was centrifuged in a Beckman Ti-70 fixed angle rotor (Analis, Gent, Belgium) at 27 000 rpm for 2 h. Fractions with a Refraction Index between 1.39 and 1.42 were pooled and further centrifuged in a Vivaspin 6 (PES, 100,000 MWCO, Sartorius AG, Goettingen, Germany) to replace iodixanol with PBS. The final sample was aliquoted and stored at −80 °C. rAAV titers were assessed by real-time PCR analysis resulting in an estimation of GC/ml using a primer probe set for the polyA sequence.

### AAV2/9 _ U6-Mical sgRNA2 exon 3-TnnT4-intron-3flag-Cre and AAV2/9 _ U6-SHAM-sgRNA TnnT4-intron-3flag-Cre injections in H11^LSL-Cas9^ mice

H11^LSL-Cas9^ CRISPR/Cas9 knock-in mice were purchased from the Jackson Laboratory (Stock No: 026816). These mice have Cre recombinase-dependent expression of CRISPR associated protein 9 (cas9) endonuclease directed by a CAG promoter. When used in combination with single guide RNAs (sgRNAs) and a Cre recombinase source, they allow editing of single or multiple mouse genes in vivo or ex vivo. For this experiment, six male and six female H11^LSL-Cas9^ mice of 5 weeks were equally divided in two groups of three males and three females, for Mical2 sgRNA treatment and sham injections. At day −2, we have performed an acute muscle damage by 10 μM solution of cardiotoxin (CTX, Naja Mossambica, Sigma-Aldrich) injected in the TA, GC and Q of the right hind limb. At day 0, 1.5 × 10^11^ genome copies of AAV2/9_U6-Mical sgRNA2 exon 3-TnT4-intron-3flag-Cre and AAV2/9_U6-SHAM-sgRNA TnT4-intron-3flag-Cre were injected in TA, GC and Q of both hind limbs, dissolved in 50 µl. Three different time points—day 10, day 21 and day 30—were set for molecular, IF and IHC analyses. Mice were sacrificed at the aforementioned time points and all the hind limb skeletal muscles together with the heart were collected for the analyses. Five time points were established for the functional test (treadmill exhaustion test), namely day 6, day 10, day 16, day 21 and day 30.

### Hematoxylin & Eosin staining

H&E staining was performed on frozen sections of TA and hearts of AAV-Mical2 H11^Cas9^ and AAV-SHAM H11^Cas9^ mice. Frozen sections were fixed in 4% PFA for 15 min, rinsed in PBS 2 min×3. Sections were stained in Harris hematoxylin solution (Sigma-Aldrich) for 4 min, followed by a washing step in running tap water for 2 min. Samples were differentiated in 1% acid alcohol for 1 min, followed by a washing step in running tap water for 1 min. The samples were put in bluing solution (Thermo Fisher) solution for 1 min and washed in running tap water for 1 min. Counterstain in eosin solution (0,1% erithrosin extra bluish Sigma-Aldrich in 70% ethanol) was done for 1 min. Dehydration steps through 95% alcohol and 2 changes of absolute alcohol for 3 min each. Lastly, samples were cleared in 2 changes of xylene, 5 min each and mounted with DPX mountant (Sigma).

### Picro-Sirius red staining

Picro-Sirius Red Stain was performed on frozen sections of TA and hearts of AAV-Mical2 H11^Cas9^ and AAV-SHAM H11^Cas9^ mice using Vitro View TM Picro-Sirius Red Stain Kit (Cat. No. VB-3017). Frozen sections were fixed in 10% formalin for 30 min, rinsed in distilled water 2 min × 3. In the meantime, Weigert’s working hematoxylin was prepared mixing Weigert’s Hematoxylin Solution A and B at 1:1 ratio. The sections were covered with Weigert’s haematoxylin for 8 min, thus to stain the nuclei and then the slides were washed for 10 min in running tap water. The samples were then stained in Picro-Sirius Red Solution for 1 h and washed in two changes of acidified water, provided by the kit. Two more washing of 2 min in dH_2_O were performed, followed by a dehydration step with 2 changes of 95% Ethanol and 2 changes of 100% Ethanol (2 min per change). Lastly, the sections were cleared with 3 changes of xylene (5 min per change) and mounted with DPX mountant (Sigma). Images were taken using an Eclipse Ti Microscope in bright-field mode and NIS-Elements AR 4.11 Software (both from Nikon). Collagen is shown in red, muscle fibers and cytoplasm are seen in yellow.

### Treadmill exhaustion test

A group of six (three males and three females) AAV-Mical2 H11^Cas9^ and six (three males and three females) AAV-SHAM H11^Cas9^ mice underwent functional tests by the treadmill exhaustion test. The test was performed at day 6, 10, 16, 21 and 30 after the beginning of the experiment (day 0). However, some of the animals were sacrificed before completing the experiments due to humane endpoints established by the Ethical committee (ECD number P162/2018). The electric shock frequency and intensity were pulses of 200 msec/pulse of electric current with 2 pulse/sec repetition rate (3 Hz) and intensity (1.22 mA), as indicated by Castro and Kuang^23^. The mice were introduced to the treadmill belt and an adaptation time of 5 min was given before the recordings (motor speed set to zero, for 5 min). A training time of 2 min at 3 m/min was set. Later on, the motor speed was set to 4 m/min, with a 1 m/min increase and a constant uphill inclination of 20°, until exhaustion and >10 s stop. The mice were weighted right after every run. Speed (m/min), distance (m) and time (min and sec) were registered and used for calculating the work of each run in J. The formula here applied was

$${\rm{Work}}\, ({\rm{J}})={\rm{body}}\, {\rm{mass}}\,({\rm{kg}}) \times {\rm{gravity}}\, (9.81{\rm{m}}/{\rm{s}}^2) \times {\rm{vertical}} \,{\rm{speed}} ({\rm{m}}/{\rm{s}} \times {\rm{angle}}) \times {\rm{time}}\, ({\rm{s}}).$$

### Transient transfection

Transient transfections were performed for both Mical2 depletion and overexpression assays in C2C12 cells and mSCs. When cells were ~50% confluent, transfections were done by using lipofectamine 2000 (Invitrogen). A solution made of Opti-MEM medium with 1:20 lipofectamine 2000 was incubate at room temperature (RT) for 5 min. In parallel, the same volume of Opti-MEM was incubated for 5 min with either 1:20 of Mical2-esiRNA and scrambled-esiRNA (Mission esiRNA Sigma Aldrich) for silencing experiments, or with 1 µg of pMical2-eGFP-N2 and eGFP-N2 overexpressing plasmid (Clontech, kindly given to our use by professor Debora Angeloni, Sant’Anna Institute, University of Pisa, Italy) for Mical2 overexpression experiments. The Opti-MEM solution with either esiRNA or plasmid was put in the lipofectamine solution and incubated together for 20 min at RT. Meanwhile, culture medium was removed and cells were washed once with PBS. The transfection solution was added to the cells and left for 9 h at 37 °C in a humidified atmosphere of 5% CO_2_. Maintenance medium was added after 9 h to stop the transfection. After one night in maintenance medium, cells could be kept proliferating, undergo differentiations or be treated with bromodeoxyuridine (BrdU) for further flow cytometry analyses.

### MLV transfer plasmid design and vector production

All viral vectors were designed and produced by the Leuven Viral Vector Core. pSRS11.EF1a.MmMical2 transfer plasmid was generated by cloning of the Mm Mical2 cDNA.

All enzymes were purchased from (Thermo Fisher Scientific; Brussels; Belgium). The integrity of all plasmids was verified by DNA sequencing. MLV-based vectors were produced as previously described by a triple polyethylenimine (PEI, Polysciences; Kampenhout; Belgium) based transfection of HEK 293T cells with pVSV-G envelope (pLP-VSV-G #646B, Thermo Fisher Scientific; Brussels; Belgium), pcDNA3.MLV.gp packaging plasmids and a SIN LTR pSRS11.SFFV.GFP transfer plasmid encoding a SFFV-driven EGFP reporter (provided by Prof. Axel Schambach^24^ or pSRS11.EF1a.Mm Mical2 transfer plasmid^25^. After filtering (0.45 µM filter—Corning Inc.; Seneffe; Belgium), vector supernatants were concentrated by tangential flow filtration using a Vivaspin (Vivascience; Bornem; Belgium). The concentrated vector was aliquoted and stored at −80 °C. Subsequently, functional transducing titers were determined in HEK 293T cells and reverse transcriptase units (RTU, non-functional titration) were measured by the SYBRGreen-I product-enhanced reverse transcriptase assay (SG-PERT). The resulting vectors are referred to as RV_SRS11-EF1a-MmMical2 and RV_SRS1-SF-eGFP.

### Generation of Mical2-overexpressing cell lines

Murine ESCs were transduced with 2.38×10^6^ titration units of RV_SRS11-EF1a-MmMical2 and RV_SRS1-SFFV-eGFP, for overexpressing Mical2 and sham control, respectively. After one cell passage, colonies were single cell resuspended and individual clones were grown and screened for Mical2 expression (see Fig. S4A, B). These cells were further used for cardiac differentiation studies.

### Flow cytometry analysis (FACS)

Fluorescence cytometry analysis (FACS) was employed for monitoring the cell cycle, after BrdU incorporation. Mical2-silenced C2C12 cells were kept proliferating for 24 h and 36 h time points. At each time point, cells were treated with 1:500 from 10 µM mouse anti-BrdU (BD Biosciences), which was incorporated in newly synthesized DNA of replicating cells. Treated cells were incubated for 1 hour at 37 °C in a humidified atmosphere of 5% CO_2_. Cells were then detached, counted and 1 × 10^5^ cells were suspended in 100 µl of appropriate culture medium. After washing the cells in 1% BSA/PBS and spinning at 500 × *g* for 15 min at RT, the pellet was resuspended in 200 µL of 1X PBS on ice. 5 mL of 70% ethanol was placed and cells were incubated on ice for 30 min to undergo fixation. The cells were then centrifuged 500 × *g* for 10 min at 10 °C. Supernatant was carefully removed and pellets were loosened by vortexing. 1 mL of 2 N HCl/Triton X-100 was added to the cells while maintaining a vortex. This step was followed by an incubation at RT for an additional 30 min, thus to denature DNA and produce single-stranded molecules. Cells were centrifuged at 500 × *g* for 10 min. The supernatant was aspirated and pellets were resuspended in 1 mL of 0.1 M Na_2_B_4_O_7_ × 10 H_2_O, pH 8.5, to neutralize the acid. Cells were centrifuged at 500 × *g* for 10 min. The supernatant was aspirated and pellets were resuspended in 1 mL of 0.5% Tween 20/1% BSA/PBS. For indirect immunostaining, 0.5 µg/ml of anti-BrdU (Sigma Aldrich) were added to the cells in a PBS-BSA 1%-tween 0.5% solution and incubated for 30 min at RT. After centrifuging for 5 min at 500 × *g*, supernatant was discarded and cells were resuspended in a PBS-BSA 1%-tween 0.5% solution with 16 µg/ml of 647 anti-mouse (Alexa Fluo) secondary antibody. They were then incubated for 30 min in the dark at RT. After washing the cells with PBS-BSA 1%-tween 0.5% and centrifuging them for 5 min at 500 × *g*, cells were incubated for 30 min in the dark at RT with 0.015 µg of propidium iodide (PI) suspended in PBS. After washing with PBS and centrifuging for 5 min at 500 × *g*, cells were resuspended in 300 µl of PBS, and filtered in FACS tubes. Lastly, cells were analyzed and quantified by flow cytometry (BD FACSCanto I or II with HTS; BD Biosciences) and FlowJo Software (FlowJo LLC) was used for data interpretation.

### Ca^2+^ imaging

Murine ESC clones #5, #6 and sham-GFP control differentiated to cardiac tissue were loaded with Cal-590 AM and Ca^2+^ imaging experiments were performed with only minor modifications to the standard procedures. In brief, cells were seeded on gelatin-coated 12-well plates before differentiation. Following 11 days from cardiac induction, cells were loaded with 1 µM of Cal-590 AM (AAT Bioquest) and 1:2000 of 20% solution in DMSO of Pluronic F-127 (Thermo Fisher Scientific) for 45 min at 37 °C and 5% CO_2_ in cardiac differentiation medium. After loading the cells de-esterification of the dye was started by replacing the medium with fresh medium lacking phenol red, to minimize interference of with the fluorescent Ca2+ indicator, for 30 min at 37 °C and 5% CO_2._ The 12-well plates were then mounted on a 37 °C heated stage (Brand and ref) of an inverted microscope (Zeiss Axio Observer Z1) equipped with a 20X air objective and a high-speed digital camera (Axiocam Hsm, Zeiss, Jena, Germany). Cal-590 was excited at the wavelength of 540 nm, and emission was detected at >590 nm. Spontaneous changes in [Ca^2+^]_i_ are expressed as *F*/*F*_0_, where *F* is the fluorescence intensity normalized to the resting fluorescence (*F*_0_).

### Statistical analysis

Statistical analyses and graphs of the results were performed on GraphPad Prism 7.0 (GraphPad Software, San Diego, CA, USA). Two-tailed *t* test or one-way ANOVA were used to compare interrelated samples. While two-way ANOVA was used to compare multiple factors. Confidence intervals were fixed at 95% (*p* < 0.05), 99% (*p* < 0.01) and 99.9% (*p* < 0.001). Data are reported as mean ± standard error of the mean (SEM). The number of independent experiments and any other specific information regarding the statistical test used and significance of the differences are specified in the figure legends.

## Results

### C2C12 and satellite cells require MICAL2 for myogenic differentiation

**Fig. 1:**
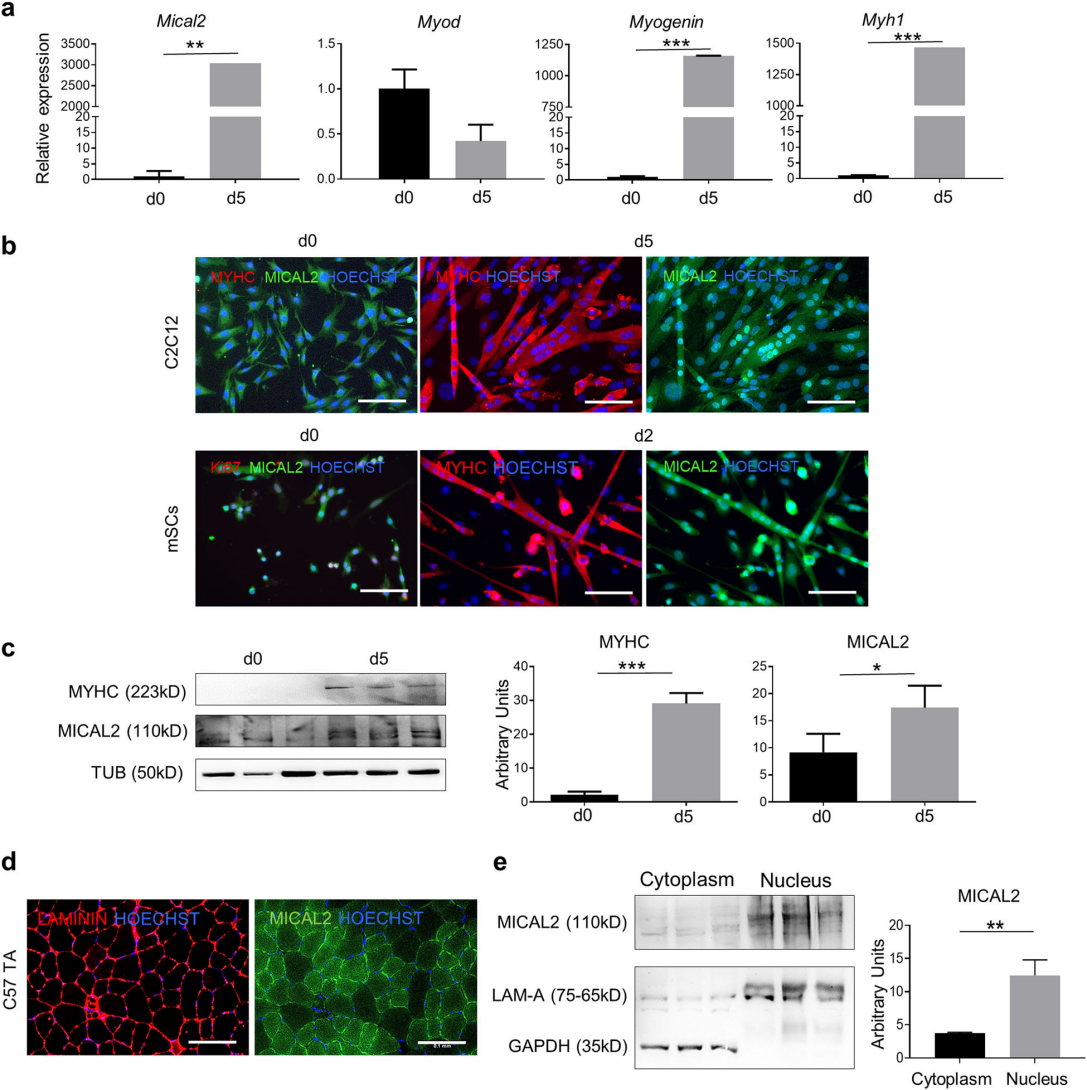
MICAL2 increases in C2C12 and mSCs differentiated myotubes. **a** qRT-PCR showing the relative expression of *MyoD, Myogenin, MyH1* and *Mical2* at day0 and day5 of C2C12 cell differentiation. **b** IF assay for MyHC (red) and MICAL2 (green) at day 0 and day 5 (C2C12) or day 2 (mSC) of skeletal muscle differentiation. Nuclei stained with HOECHST (blue). Scale bars 200 μm. **c** WB for MyHC and MICAL2 proteins on proliferating (day 0) and differentiating (day 5) C2C12 cells. The relative quantification is on the right. **d** IF analysis for MICAL2 (green) and LAMININ (red) localization on TA cross-sections of C57/Bl6 (C57). Scale bars 100 μm. *N* = 3. **e** WB for MICAL2 cytoplasmic and nuclear protein fractions on C57/Bl6 (C57) GN. The relative quantification is on the right. * = *p* < 0.05; ** = *p* < 0.01; *** = *p* < 0.001 by two-tailed *t* test. See also Figs. S1–S3.

We have differentiated C2C12 cells and mSCs—isolated from C57/Bl6 mice—towards skeletal muscle to determine MICAL2 presence in differentiated cells, compared to their progenitors. In this regard, analyzing the RNA expression of successful differentiations—proven by *MyoD, Myogenin* and *MyH1* as myogenic markers—*Mical2* has been found significantly increased after myotubes formation (Fig. 1a). IF analysis has shown MICAL2 localization in both proliferating and differentiated C2C12 cells and mSCs. While MICAL2 is distributed in both cytoplasm and nuclei during proliferation (d0), MICAL2 has a predominant nuclear localization in differentiating myotubes (d5 and d2, respectively), in both cellular types (Fig. 1b). Protein analysis has revealed that MICAL2 is increased in differentiated cells (d5) compared to proliferating cells (d0) (Fig. 1c). These results were confirmed at a visual level by staining C57/Bl6 TA sections for MICAL2. As depicted, MICAL2 has a cytoplasmic and nuclear localization in steady state muscles (Fig. 1d). Moreover, we confirmed MICAL2 distribution by a cytoplasmic-nuclear protein separation of C57/Bl6 gastrocnemius (GN), in which MICAL2 is present in both compartments but mainly in the nuclei (Fig. 1e). Therefore, we indicate here that MICAL2 is present and increases during skeletal muscle differentiation. Further proof of MICAL2 importance in myogenesis has been evaluated in loss and gain of function studies of these two cellular types (Supplementary Figs. 1–3). After Mical2-esiRNA administration to cultured cells, we have achieved a transient MICAL2-knockdown in C2C12 cells and mSCs, of which we have estimated the efficiency (Supplementary Fig. 1a). The impact of MICAL2 on proliferation was evaluated by looking at the cell cycle. BrdU incorporation in C2C12 cells was analyzed by flow cytometry 24 h and 36 h after transfection. A higher number of cells is gathered in the S phase of MICAL2 silenced cells compared to lipofectamine treated cells (22% vs 15% and ~21% vs ~12% at 24 h and 36 h, respectively), meaning that cells lacking MICAL2 were more prone to proliferation (Supplementary Fig. 1b). The increased proliferation was also observed by immunostaining for the nuclear proliferation marker Ki67 in C2C12 cells and mSCs. Indeed, in both MICAL2-silenced cell types the ratio between Ki67+ nuclei and total number of nuclei is significantly higher for MICAL2 silenced cells compared to lipofectamine treated cells (Supplementary Fig. 1c). Lastly, phosphorylated ERK (P-ERK)—Mitogen-Activated Protein Kinase (MAPK) ^26^—has been evaluated since it is known to regulate muscle growth and function. The results indicated that protein ratio between P-ERK and total ERK is higher in MICAL2-silenced cells and gradually decreased overtime in cells recovering from silencing (Supplementary Fig. 1d).

After having assessed that MICAL2 silencing had an effect on proliferation, we elucidated whether this knockdown also had an impact on myotube formation. C2C12 cells and mSCs silenced cells underwent skeletal muscle differentiation for 5 days and 2 days, respectively (see Methods). Intriguingly, MICAL2 silencing has restrained cells from fusing and forming myotubes. Indeed, MICAL2 silenced cells do not express MyHC protein and do not form fused myotubes in culture, compared to differentiated lipofectamine treated cells (Supplementary Fig. 2a). This experiment has been repeated five times and fusion index (FI) was calculated as ratio of two or more MyHC+ nuclei within myocytes versus the total number of nuclei, reported as percentage. FI has shown a significant difference between MICAL2-silenced cells and lipofectamine treated cells, with very high numbers for the former and almost null for the latter. Additionally, mSCs have confirmed these data (Supplementary Fig. 2b). Moreover, skeletal muscle differentiated mSCs were checked for Ki67 proliferation marker and their relative ratio between Ki67+ nuclei and total number of nuclei has indicated that more silenced cells were Ki67+ compared to lipofectamine treated cells (Supplementary Fig. 2b). MyHC protein quantification has corroborated the aforementioned result showing in lipofectamine treated cells with more abundant MyHC content, compared to MICAL2-silenced cells (Supplementary Fig. 2c). In conclusion, MICAL2 depletion led to an impairment of skeletal muscle differentiation and the exposure to Mical2esiRNA treatment resulted in a detrimental effect on C2C12 cells and mSCs myogenic differentiation compared to controls.

In a next step, we aimed to assess whether MICAL2 overexpression was able to affect myogenic differentiation. C2C12 cells were transiently transfected to express MICAL2 (see Methods) and soon after placed in skeletal muscle differentiation medium for 5 days—after being tested for their efficiency (Supplementary Fig. 3a). Analyzing MyHC protein content by IF unveiled thicker myotubes in MICAL2-overexpressing cells compared to lipofectamine treated cells (Ctrl Lipo), as well untreated cells (Ctrl), which was confirmed by FI percentage quantification (Supplementary Fig. 3b). However, MICAL2 protein distribution was similar in all samples at d5 of the differentiation protocol, possibly due to the transient effect of overexpression lasting less than 5 days. Lastly, a significant higher amount of MyHC protein is present in MICAL2-overexpressing cells compared to lipofectamine treated control cells (Supplementary Fig. 3c). In conclusion, MICAL2 gain of function analyses have enlightened a positive effect upon skeletal muscle differentiation, showing more pronounced myotube formation with more MyHC+ myotubes.

### MICAL2 increases during smooth muscle differentiation from progenitors

**Fig. 2:**
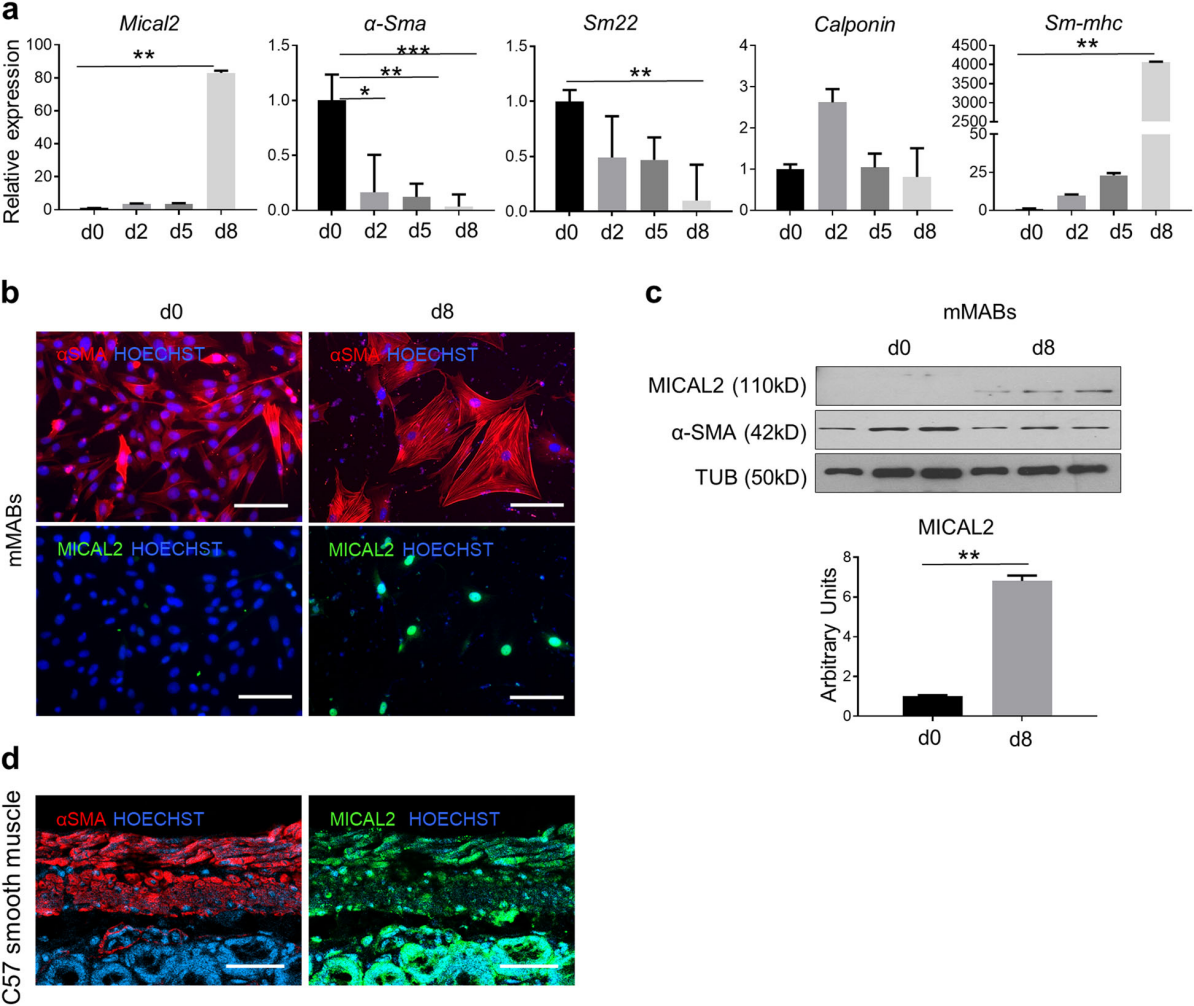
MICAL2 characterization in mMABs smooth muscle differentiation. **a** qRT-PCR showing the relative expression of *α-Sma, Sm22, Calponin, SM-MyHC*, and *Mical2* at day 0, day 2, day 5 and day 8 of smooth muscle differentiation. **b** IF assay for α-Smooth Muscle Actin (α-SMA; red) and MICAL2 (green). Nuclei stained with HOECHST (blue). Scale bars 50 μm. **c** WB and relative quantification for MICAL2 and α-SMA, on proliferating (day 0) and differentiating (day 8) mMABs. *N* = 3. **d** IF analysis for α-SMA (red) and MICAL2 (green) localization on gastrointestinal tract cross-sections of C57/Bl6 (C57). Scale bars 50 μm. *N* = 3. * = *p* < 0.05; ** = *p* < 0.01; *** = *p* < 0.001 one-way ANOVA test and two-tailed *t* test.

We differentiated murine mesoangioblasts (MABs) towards smooth muscle cells to characterize MICAL2 from progenitor to differentiated cells. We show that in a smooth muscle differentiation protocol lasting for 8 days, differentiation markers are in line with the expected maturation of the cells^27,28^. Indeed, smooth muscle differentiation genes, like *α-Smooth Muscle Actin* (*α-Sma*), *Sm22*, *Calponin* and *smooth-muscle MyHC* (*sm-MyHC*) display a typical expression pattern (Fig. 2a). *Mical2* mRNA showed steep increase in expression at the d8 time point, which coincided with nuclear MICAL2 localization by IF analysis in α-SMA positive differentiated mMABs (Fig. 2b). Similar results were obtained by WB analysis and the relative quantification, confirmed that MICAL2 protein content increased in differentiated cells (Fig. 2c). A representative ex vivo smooth muscle staining confirmed MICAL2 nuclear localization together with cytoplasmic signal in the gastrointestinal (GI) tract of C57/Bl6 mice (Fig. 2d). All together, these results show that MICAL2 is highly expressed in nuclei of differentiated smooth muscle cells and in both nuclei and cytoplasm of murine GI tract.

### MICAL2 improves cardiac differentiation of murine embryonic stem cells (mESCs)

**Fig. 3:**
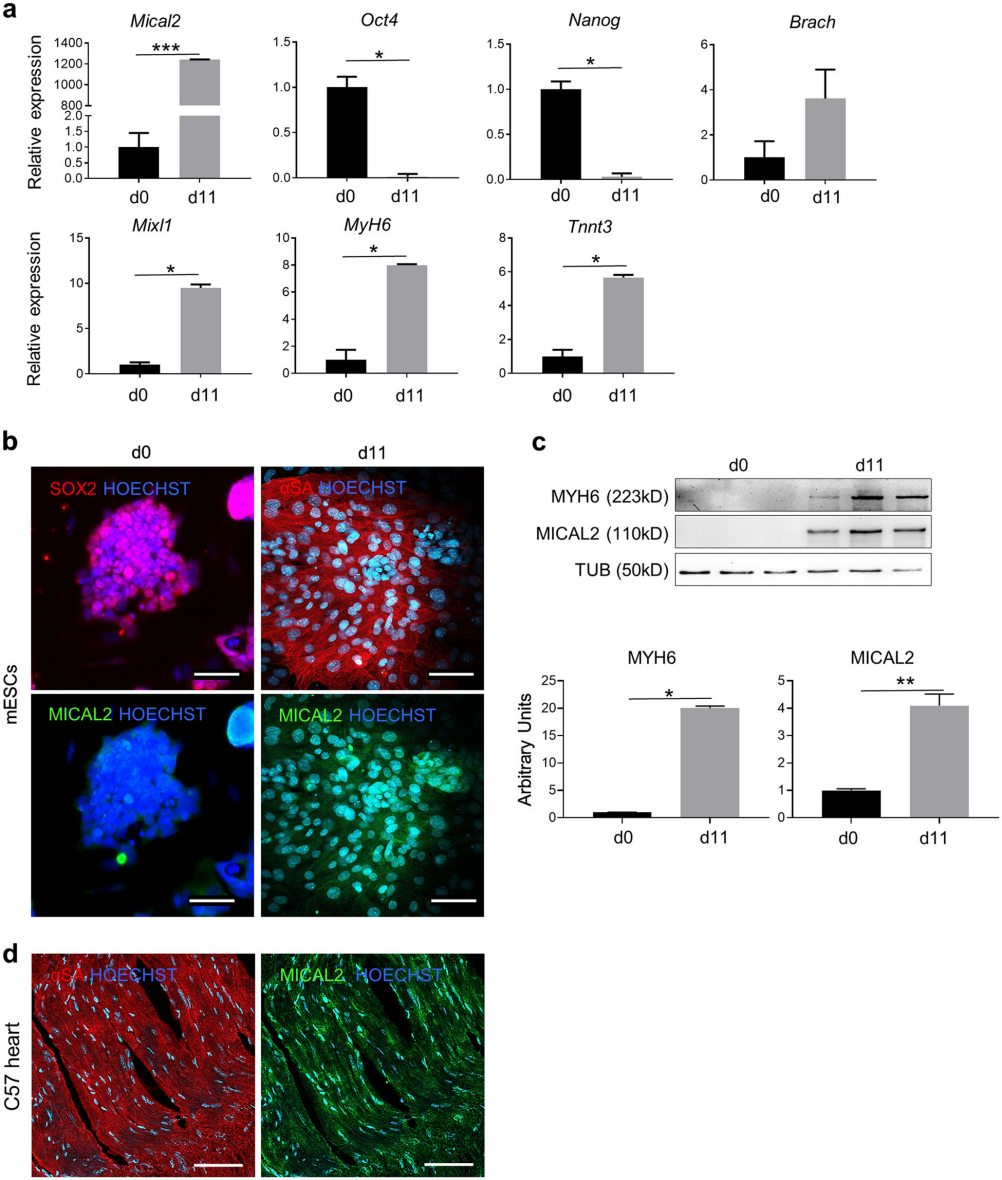
MICAL2 characterization in mESCs differentiated to cardiomyocyte-like cells. **a** qRT-PCR showing the relative expression of pluripotency markers *Oct4, Nanog*, mesoderm and progenitor markers *Brach, MixL1*, cardiac differentiation markers *MyH6* and *TnnT3*, and *Mical2* in proliferating mESCs (day 0) and in beating cardiomyocyte-like cells (day 11). **b** IF assay in mESCs at d0 for SOX2 (red) and MICAL2 (green) and day 11 of cardiac differentiation for α-SA (red) and MICAL2 (green). Nuclei stained with HOECHST (blue). Scale bars 200 μm. **c** WB and relative quantification for MyH6 and MICAL2 on proliferating (day 0) and differentiating (day 11) mESCs. *N* = 3. **d** IF analysis for α-SA (red) and MICAL2 (green) localization on heart cross-sections of C57/Bl6 (C57). Scale bars 100 μm. * = *p* < 0.05; ** = *p* < 0.01 by two-tailed *t* test. See also Figs. S4, S5.

Murine ESCs were differentiated in 11 days into beating cardiomyocyte-like cells using a differentiation protocol starting from EB aggregation (day 0). Gene expression of key transcription factors from pluripotency until cardiomyogenic differentiation, including *Nanog, Oct4, Brachyury (Brach), MixL1, cardiac-Myosin heavy chain (Myh6)* and *TnnT3*, were analyzed by qRT-PCR (Fig. 3a). Together with the aforementioned cardiac markers, we found high *Mical2* expression in mature cardiomyocyte-like cells (Fig. 3a), which was corroborated by IF analysis that showed MICAL2 and SOX2 or sarcomeric α-actinin (α-SA) sub-cellular localization in both proliferating and differentiated mESCs, respectively (Fig. 3b). Indeed, MICAL2 was almost undetectable in the pluripotency stage—compared to differentiated cells (d11)—and was predominantly nuclear in cardiomyocyte-like cells. In parallel, WB analysis and the relative quantification confirmed that MICAL2 protein is substantially expressed in differentiated cells positive for MyH6 (Fig. 3c).

Next, cardiac tissue of C57 mice has been stained for MICAL2 and α-SA showing MICAL2 localization both in the cytoplasm and in the nuclei, as in the differentiated cells (Fig. 3d). In this regard, we decided to overexpress MICAL2, thus to prove whether the cardiac differentiation was affected by MICAL2 modulation. We generated murine ES cell lines stably expressing *Mical2* following transduction with stably integrating MLV-based viral vectors RV_SRS11-EF1a-MmMICAL2 or control RV_SRS11-SFFV-eGFP vector (see Methods). After having screened for the stable Mical2-overexpressing cell lines we have chosen the mESC line #5 and #6 as the ones of our use, since both had MICAL2 gene and protein upregulated compared to sham controls (transduced with RV_SRS11-SFFV-eGFP) (Supplementary Fig. 4a, b). Following induction of cardiac differentiation, the fluorescence intensity histogram, IF and WB indicated more aSA and MyH6 proteins in Mical2-overexpressing cell lines, compared to their sham controls (Supplementary Fig. 4c, d). This cardiomyocyte-like mature phenotype was confirmed by spontaneous cytosolic Ca^2+^ events assessed in Cal-590 loaded murine ES cell lines overexpressing Mical2 (Supplementary Fig. 5a). Indeed, spontaneous Ca^2+^ events occurred with a higher frequency in MICAL2-overexpressing lines compared to controls. Moreover, cardiomyocyte-like cells from #5 (Video S1) and #6 (Video S2) Mical2-mESC lines beat more rapidly (average of ~60 bpm) than Sham controls (Video S3, ~20bpm) (Supplementary Fig. 5b).

All together, these results suggested a basal MICAL2 expression in proliferating cells that significantly increased upon cardiac induction, where MICAL2 localized mainly in cardiomyocyte-like nuclei. Moreover, MICAL2 overexpression led to a better maturation and function of cardiomyocyte-like cells.

### MICAL2 actively participates to acute and chronic regeneration in skeletal muscles

**Fig. 4:**
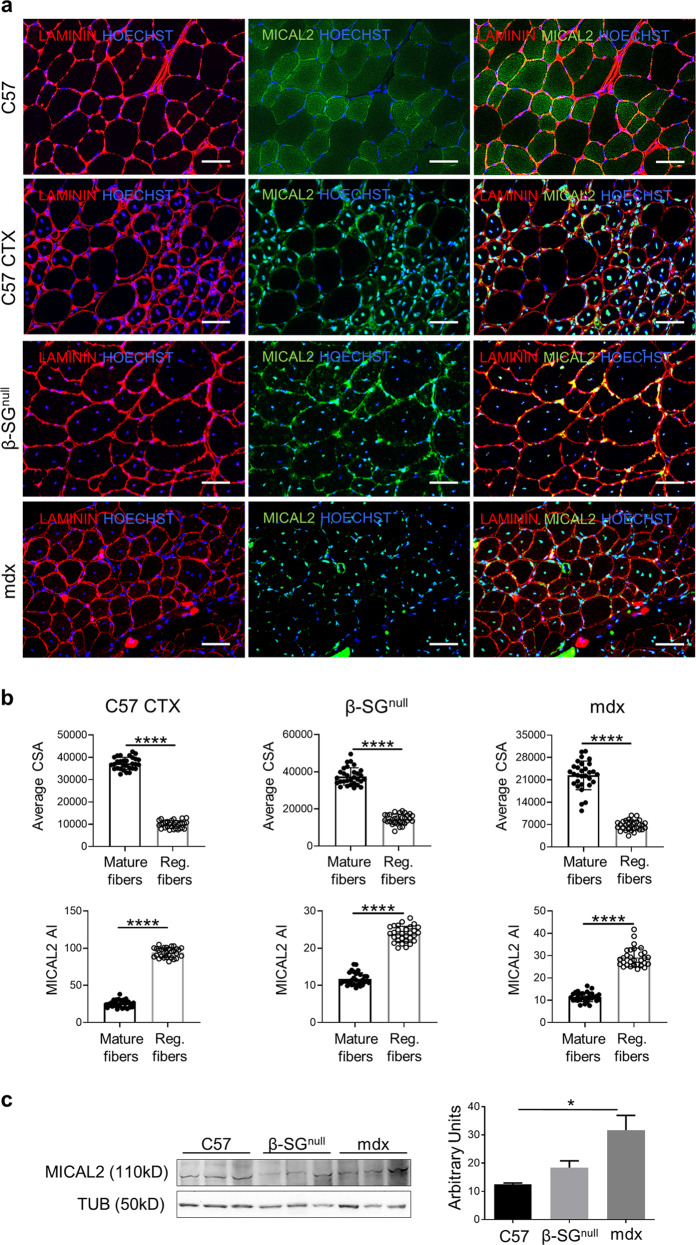
MICAL2 characterization in muscle fibers. **a** Upper panels show IF analysis for LAMININ (red) and MICAL2 (green) localization on TA cross-sections of uninjured control C57/Bl6 (C57), acute regeneration of C57/Bl6 after cardiotoxin injection (C57 CTX), chronic regeneration of β-SG^null^ and mdx dystrophic models. Nuclei stained with HOECHST (blue). Magnification 20x. Scale bars 50 μm. **b** Upper panels show average CSA between mature and regenerating fibers of C57 CTX, β-SG^null^ and mdx dystrophic models. Lower panels show MICAL2 fluorescence intensity calculated as absolute intensity (MICAL2 AI) in mature and central nucleated regenerating fibers of C57 CTX, β-SG^null^ and mdx dystrophic models. *N* = 5. **c** WB and relative quantification for MICAL2 on *gastrocnemius* of uninjured control C57/Bl6 (C57) and chronic regeneration of β-SG^null^ and mdx dystrophic models. *N* = 3. * = *p* < 0.05; **** = *p* < 0.0001 by one-way ANOVA test.

Steady state TA of C57/Bl6 mice were compared to snake venom-derived cardiotoxin (CTX) injected C57/Bl6 mice, to Sarcoglycan-β null (β-SG^null^, animal model with C57/Bl6 background resembling LGMD type 2E) mice and to mdx thus to characterize the presence of MICAL2 during acute and chronic degeneration/regeneration processes, respectively. IF experiments indicate increased MICAL2 protein in all acute and chronic degenerating/regenerating muscles—C57 CTX, β-SG^null^ and mdx TA—compared to uninjured C57/Bl6 control (Fig. 4a). This happened particularly in centrally nucleated fibers, where F-actin is constantly recruited in the regeneration process. Indeed, MICAL2 fluorescence intensity—calculated as absolute intensity (AI)—is significantly increased in central nucleated fibers with small cross sectional areas (CSA) in comparison with mature fibers in all the regenerating models here evaluated (CTX C57, β-SG^null^ and mdx (Fig. 4b). Hence, this further demonstrates the importance of MICAL2 during the regeneration process. Accordingly, the WB assay confirmed that MICAL2 was more abundant in pathologic muscles, which let us conclude that acute and chronic muscle regeneration recruited more MICAL2 in regenerating nuclei, compared to healthy control muscles (Fig. 4c).

### Cas9–Mical2 guide RNA delivery in H11^LSL-Cas9^ mice induces skeletal muscle degeneration, inflammation and fibrosis

**Fig. 5:**
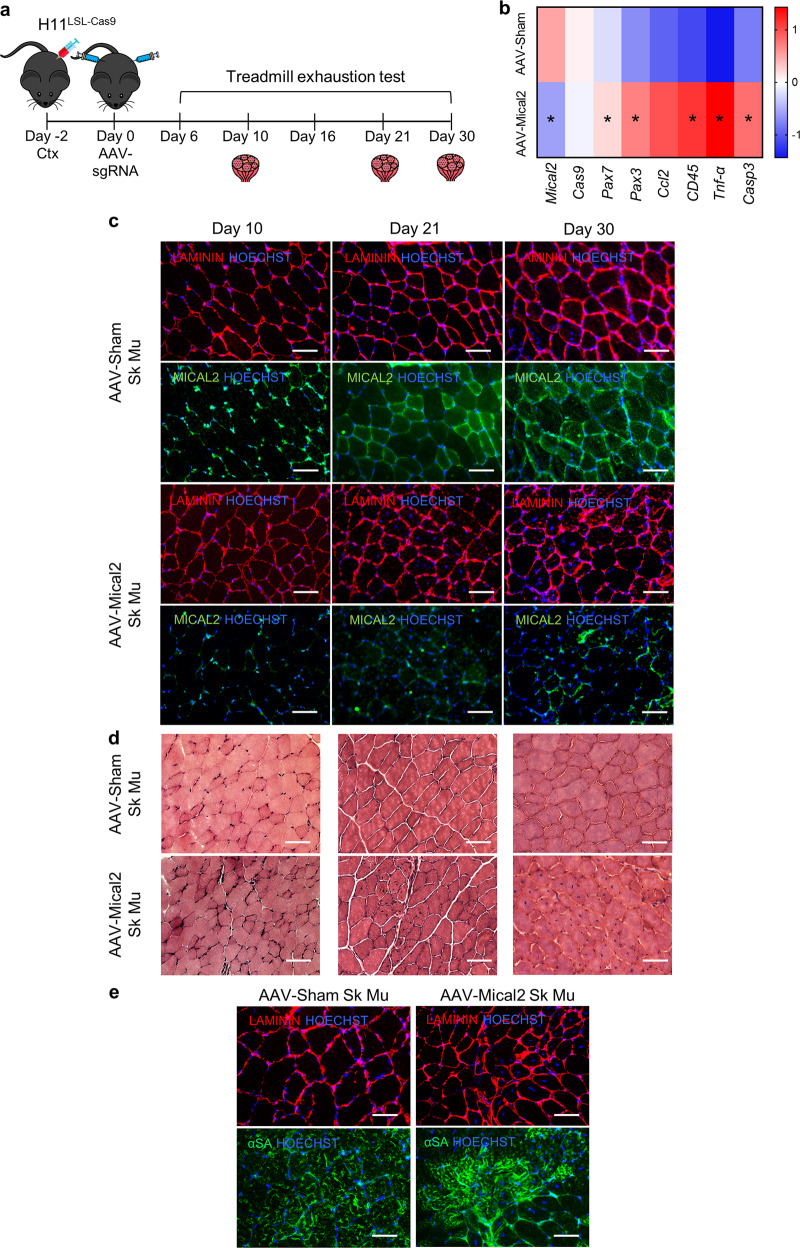
In vivo MICAL2 CRISPR/Cas9 system induces skeletal muscle degeneration/regeneration, inflammation and fibrosis. **a** Timeline illustrating the experiment setup. At day -2, H11^LSL-Cas9^ underwent skeletal muscle damage by CTX injections on the right hind limb (TA, GC and Q). At day 0, CRISPR/Cas9 system activation via AAV-Cre with either Sham sgRNA or Mical2 sgRNA in both hind limbs (TA, GC and Q). At day 6, 10, 16, 21 and 30 treadmill exhaustion tests were performed and at day 10, 21 and 30 mice were sacrificed for molecular and histological analyses. **b** Heat map representing qRT-PCR Z-score of *Mical2* and *Cas9* for the system activation, *Pax7*, *Pax3* and *Ccl2* for skeletal muscle regeneration and remodeling and *CD45*, *Tnf-α* and cleaved *Casp3* (*Casp3*) for inflammation and apoptosis. **c** IF analysis illustrates at day 10, day 21 and day 30 LAMININ (red) and MICAL2 (green) localization on left TA cross-sections of AAV-Sham H11^Cas9^ mice compared to AAV-Mical2 H11^Cas9^ mice. Nuclei stained with HOECHST (blue). Scale bars 50 μm. **d** The effect of MICAL2 CRISPR/Cas9 is shown by H&E staining on left TA cross-sections of AAV-Sham H11^Cas9^ mice compared to AAV-Mical2 H11^Cas9^ mice, along time points of 10, 21 and 30 days. Scale bars 50 μm. **e** IF analysis of LAMININ (red) and alpha-SARCOMERIC ACTININ (α-SA; green) in AAV-Mical2 muscles showed actin filament disorganization compared to controls at day 30 from infection. Scale bars 50 μm. *N* = 6. * = *p* < 0.05 by two-tailed *t* test. See also Fig. S6.

**Fig. 6:**
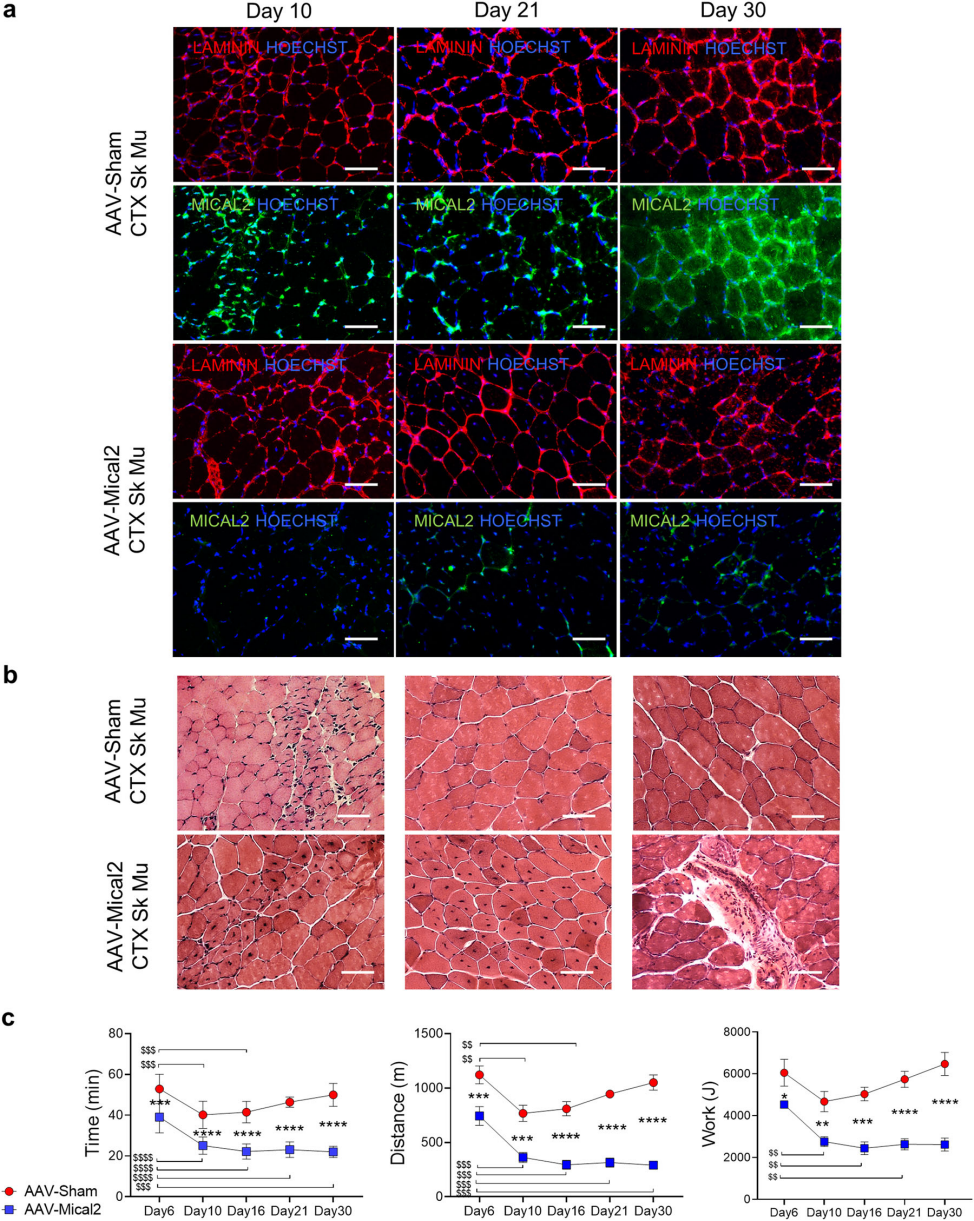
In vivo MICAL2 CRISPR/Cas9 system restrains regeneration in injured skeletal muscle and impairs muscle functionality. **a** IF analysis illustrates at day 10, day 21 and day 30 LAMININ (red) and MICAL2 (green) localization on right TA cross-sections of AAV-Sham H11^Cas9^ mice compared to AAV-Mical2 H11^Cas9^ mice, regenerating after CTX injections. Nuclei stained with HOECHST (blue). Scale bars 50 μm. **b** The effect of MICAL2 CRISPR/Cas9 is shown by H&E staining on regenerating right TA cross-sections of AAV-Sham H11^Cas9^ mice compared to AAV-Mical2 H11^Cas9^ mice, along time points of 10, 21 and 30 days. Scale bars 50 μm. **c** Treadmill exhaustion test of AAV-Sham H11^Cas9^ mice compared to AAV-Mical2 H11^Cas9^ mice along time, from the CRISPR/Cas9 activation up to one month follow up. From left to right, graphs of Time of run (min), Distance (m) and Work (J) at day 6, day 10, day 16, day 21 and day30. *N* = 6. *** = *p* < 0.001; **** = *p* < 0.0001intergroups by *t* test. $$ = *p* < 0.01; $$$ = *p* < 0.001; $$$$ = *p* < 0.0001 intragroup by two-tailed *t* test. See also Fig. S6.

To gain insight into the effects of MICAL2 downregulation on skeletal muscles, we employed H11^LSL-Cas9^ mice that in combination with a single guide RNA (sgRNA) and a Cre recombinase source allow gene editing. Thus, we generated Adeno-associated viruses (AAV Serotype2/9) which express Cre recombinase under the control of TnnT4 promoter and a single guide RNA (sgRNA) against Mical2 (AAV-Mical2) or a non-targeting sgRNA control (AAV-Sham). The timeline of our experiment began with CTX injections on *tibialis anterior* (TA), *gastrocnemius* (GC) and *quadriceps* (Q) of the right hind limb, and at 10 days from the CTX treatment, muscles show ~70% of centrally nucleated fibers (Supplementary Fig. 6a). Then, H11^LSL-Cas9^ mice were infected with AAV-Mical2 or AAV-Sham in both hind limbs (Fig. 5a). Treadmill exhaustion tests were performed, mice were sacrificed at different time points and the skeletal and cardiac muscles dissected for further analyses (Fig. 5a). The heat map representing qRT-PCR Z-scores showed *Pax7*, *Pax3* and *Ccl2* and *CD45*, *Tnf-α* and cleaved *Casp3* (*Casp3*) upregulated in AAV-Mical2 muscles compared to AAV-Sham muscles (Fig. 5b). LAMININ and MICAL2 protein localization shown by the IF analysis indicated AAV-Mical2 and AAV-Sham infected muscles at day 10, day 21 and day 30 (Fig. 5c). The reduced signal of MICAL2 protein was evident in all time points of AAV-Mical2 muscles and quantified by WB as a MICAL2 reduction of ~77% (Supplementary Fig. 6b). At day 21 was visible fiber degeneration and regeneration, muscle fibrosis accompanied by a large increase in muscle fiber size variation (Fig. 5d) by H&E and supported by Picro-Sirius red staining (Supplementary Fig. 6c). Interestingly, the lack of MICAL2 in uninjured skeletal muscle fibers led to a diffuse disorganization of ACTIN filaments resulting in ACTIN defects within skeletal muscle fibers (Fig. 5e). These actin defects were already demonstrated in Drosophila^14^. The intramuscular injection of CTX—which induces myolysis and triggers the regeneration process—exacerbated the effect of reduced amount of MICAL2 proteins, as shown by histological analyses (Fig. 6a, b and Supplementary Fig. 6e) and corroborated by protein quantification (Supplementary Fig. 6d), which showed 78% of MICAL2 reduction. Moreover, in both uninjured and injured skeletal muscle, there was a decrease of MYHC protein in AAV-Mical2 treated mice (Supplementary Fig. 6b, d). Finally, the treadmill exhaustion test provided evidence that the reduced amount of MICAL2 in both hind limbs compromised muscle performance, as the time of running, distance and work (Fig. 6c).

### Cas9–Mical2 guide RNA delivery in H11^LSL-Cas9^ mice induces cardiac muscle degeneration, inflammation and fibrosis

**Fig. 7:**
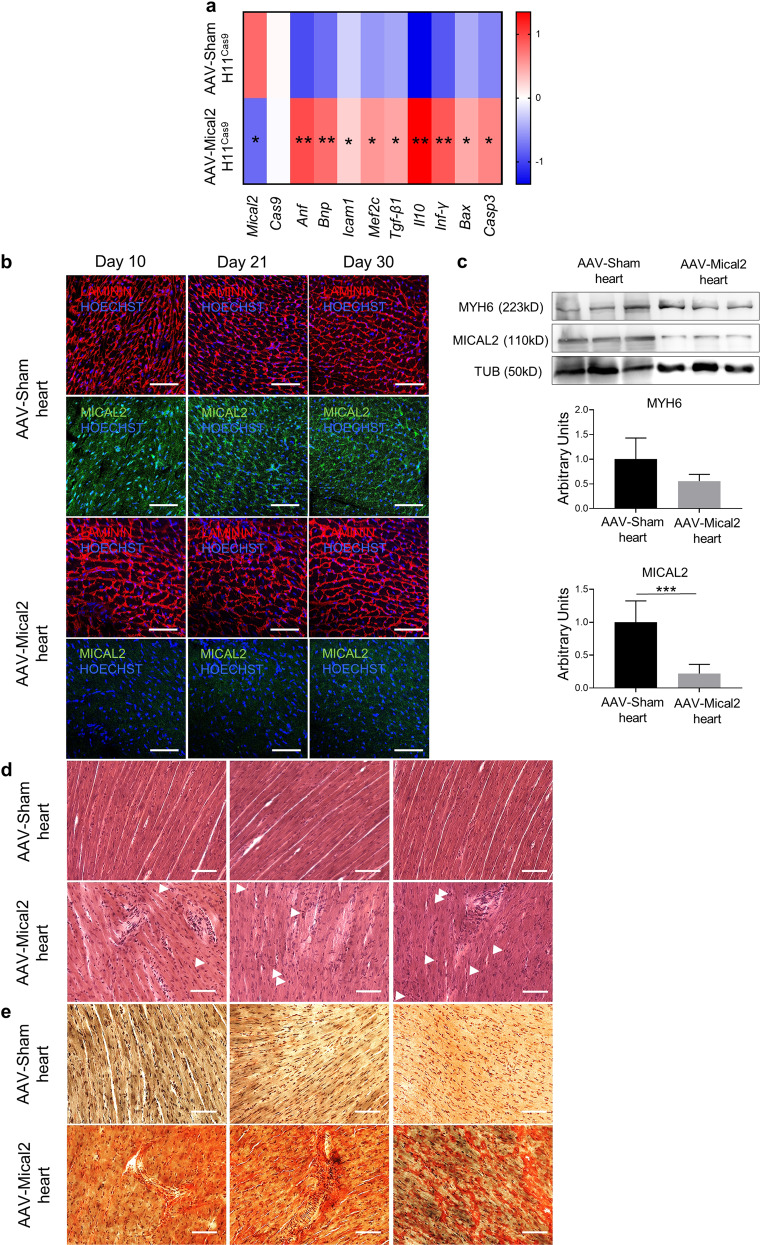
In vivo MICAL2 CRISPR/Cas9 system induces cardiac muscle remodeling, inflammation, fibrosis and apoptosis. **a** Heat map representing qRT-PCR Z-score of *Mical2* and *Cas9* for the system activation, *Anf* and *Bnp* for cardiac stress, Mef2c, Tgf-β and Il10 for cardiac remodeling and fibrosis, *Icam1* and *Ifn-γ* for cardiac inflammation and *Bax* and cleaved *Casp3* (*Casp3*) apoptosis. **b** IF analysis illustrates at day 10, day 21 and day 30 LAMININ (red) and MICAL2 (green) localization on heart cross-sections of AAV-Sham H11^Cas9^ mice compared to AAV-Mical2 H11^Cas9^ mice. Nuclei stained with HOECHST (blue). Scale bars 100 μm. **c** WB for MYH6 and MICAL2 proteins on hearts of AAV-Sham H11^Cas9^ mice compared to AAV-Mical2 H11^Cas9^ mice. The relative quantification is below. *N* = 3. **d** The effect of MICAL2 CRISPR/Cas9 is shown by H&E staining on heart cross-sections of AAV-Sham H11^Cas9^ mice compared to AAV-Mical2 H11^Cas9^ mice, along time points of 10, 21 and 30 days. Scale bars 50 μm. **e** Collagen deposition and fibrosis (red) due to the effect of Mical2 CRISPR/Cas9 are shown by Picro-Sirius red staining on heart cross-sections of AAV-Sham H11^Cas9^ mice compared to AAV-Mical2 H11^Cas9^ mice, along time points of 10, 21 and 30 days. Scale bars 50 μm. *N* = 6. * = *p* < 0.05; ** = *p* < 0.01; *** = *p* < 0.001 by two-tailed *t* test.

Within the rodent cardiac system, AAV 9 serotype has demonstrated cardiac tropism and is particularly efficient in cardiac transduction via the transvascular route. Thus, we investigated the effect of AAV-Mical2 treatments in cardiac tissue in comparison with AAV-Sham treated mice. The heat map representing qRT-PCR Z-scores showed *Anf*, *Bnp, Icam1*, *Tgfβ1, Il10, Inf-γ, Bax* and cleaved *Casp3* (*Casp3*) upregulated in AAV-Mical2 treated mice compared to AAV-Sham treated mice (Fig. 7a). LAMININ and MICAL2 cardiac localizations shown by IF analysis at day 10, day 21 and day 30 from treatments highlighted the reduced signal of MICAL2 protein in all time points of AAV-Mical2 cardiac tissues (Fig. 7b). MICAL2 protein reduction has been quantified as 78% in the heart of AAV-Mical2 mice (Fig. 7c). In addition, in all time points analyzed, foci of cardiac degeneration were visible, accompanied by muscle fibrosis as shown by H&E (Fig. 7d) and supported by Picro-Sirius red staining (Fig. 7e). Thus, AAV-Mical2 treatment negatively affects cardiac tissue in H11^LSL-Cas9^ mice.

## Discussion

Despite the importance of actin remodeling in myogenic differentiation little is known regarding nuclear monooxygenase that promotes depolymerization of F-actin. This is mainly due to the activity of MICAL2 that mediates oxidation of methionine in order to reversibly depolymerize the actin filament. In addition, MICAL2 is implicated in the remodeling of mdx muscles, as reported in a genome-wide screening^20^. Thus, to fill the gap between in silico analyses and speculative hypotheses we investigate the role of MICAL2 in myogenic differentiation using cell and transgenic animal models and employing an overexpression and CRISPR-mediated Mical2 depletion approach. Taken together, our results demonstrate that MICAL2 increases its expression in the three muscle lineages from adult progenitor cells (mSCs, C2C12 cells and mMABs), or pluripotent cells (mESCs and hiPSCs). Serum response factor (SRF) is a transcription factor common also to the three muscle lineages^29^ and that based on its interactions it can either modulate skeletal, smooth and cardiac muscle commitments and functions^30–33^. Not surprisingly, SRF is tightly regulated by MICAL2^11^. Indeed, when MICAL2 depolymerizes F-actin it causes G-actin export from nuclei, determining myocardin-related transcription factor-A (MRTF-A) accumulation in the nucleus. Therefore in this way, SRF can bind MRTF-A promoting the activation of several genes^11^ in smooth and cardiac muscle regulation^34–36^.

Differently from MICAL2 and SRF, specific genes are peculiar to a single muscle lineage and their activation only leads to one muscle commitment. While MyoD and MyoG are crucial only for skeletal muscle commitments^37^, GATA4, TBX5 and NKX2.5 regulate cardiac differentiation^38,39^. TGF-β, instead, can activate several signaling pathways such as RhoA, Notch and SRF/myocardin leading to smooth muscle cell fate determination and maturation^40^. However, it is shown that regulating one of the aforementioned cross-talks could induce lineage promiscuity in myogenic progenitors. An example is the expression of miR669a and miR669q, which is known for negatively regulating MyoD activation^41^. In LGMD-2E, cardiac progenitors undergo aberrant skeletal muscle differentiation and this was found to be due to the absence of miR669a and miR669q in those cells, causing a switching from cardiac to skeletal muscle commitment. Since MICAL2 regulation is important for both striated muscles, it may be interesting to investigate its interaction with MyoD and miR669 family and its role in this aberrant phenotype.

MICAL2 locates to the nucleus where it acts as redox enzyme. It is known that MICAL1 and MICAL3 carry a coil-coil domain able to auto inhibit their cellular activities when they are in the cytoplasm^19^. However, such domain is not present in MICAL2 and its auto-inhibition mechanism is not yet known, although it is not active in the cytoplasm^19^. We showed here that MICAL2 resides in the cytoplasm of myogenic progenitors (mSCs and C2C12 cells) and it translocates into nuclei when the cells differentiate towards skeletal muscle. The nuclear localization is also shown in smooth muscle differentiated mMABs and in cardiomyocyte-like cells originated from PSCs. In addition, MICAL2 is only present at low levels in the cytoplasm of healthy muscle fibers, while in acute and chronic degeneration/regeneration conditions it is highly expressed in the regenerating nuclei. Thus, MICAL2 shuttles between cytoplasm and nucleus to maintain cellular homeostasis, in line with the well-known YAP-TAZ system^42,43^. Similarly to MICAL2, YAP/TAZ nuclear localization occurs when cardiomyocyte progenitor cells differentiate towards cardiac-like cells^44^. Moreover, YAP/TAZ shuttling is activated in response to dynamic modifications and seems to be required for cardiac progenitor cell motility on stiff surfaces^44^. Indeed, cell migration is another important effect that might involve MICAL2 action as documented in previous studies^45,46^, and epithelial to mesenchymal transition (EMT) studies fully support this hypothesis^45^. MICAL2 was addressed as a regulator of EMT and the reverse process mesenchymal to epithelial transition (MET) in several cancer types. Particularly, high expression of MICAL2 correlates with EMT and thus with cell migration, whereas MET occurs when MICAL2 is turned off, or decreased in cancer cells^19,45,46^. It is also likely that MICAL2 may have an impact in non-tumor cell migration since depolymerization/repolymerization cycles are required for cell rolling. This could be relevant also for SCs, C2C12 cells^47^, MABs and mesodermal progenitors^48,49^ that are known to have migration capacity. Further investigation using scratch and invasion assay are necessary to understand whether MICAL2 is involved in cell migration of non-tumor cells.

Taken together, our observation regarding MICAL2 expression under physiological conditions is not in line with the literature about cancers. Indeed, high MICAL2 protein levels correlate with high proliferation in several tumors^19,45,46^, while MICAL2 is expressed at higher levels in differentiated myogenic cells—in all the three lineages—compared to their progenitors. Moreover, Mical2 overexpression showed more skeletal muscle differentiation in C2C12 cells. In addition, we observed an increased proliferation and a consequent lack of differentiation in C2C12 cells and mSCs after silencing of MICAL2. These results need deeper investigations to shed light on the discrepancy between the cancer field and muscle physiology. For this reason, we are currently investigating the role of MICAL2 in different rhabdomyosarcoma cell lines. However, in our findings, MICAL2 is highly expressed in acute and chronic degeneration/regeneration processes and the reason why this high expression occurs is not clear yet. In the dystrophic situation there is a constant regeneration going on, in which myogenic cell progenitors try to reconstitute the myogenic pool^50,51^. Thus, we hypothesize MICAL2 may be an important therapeutic target in these pathological conditions. Next, we combine the CRISPR/Cas9 system into the skeletal muscle biology using AAV and transgenic approaches. This system enables the rapid functional investigation of Mical2 gene inactivation in adult muscle tissue without the efforts to generate an *ad hoc* colony of transgenic mice, thus in line with 3R policy to reduce animal use in biomedical research. The reduced amount of MICAL2 in hind limbs compromises skeletal muscle biology of AAV-Mical2 H11^Cas9^ mice that negatively affects muscle performance, as indicated by treadmill exhaustion tests. However, due to the AAV-Mical2 high transduction efficiency, also cardiac tissue is affected and it is likely that reduced cardiac function contributes to the observed skeletal muscle phenotype. We believe that sgRNA-directed Cas9 cutting should also enable the rapid generation of complex structural genomic alterations, inserting multiple sgRNAs on viral vectors would allow the inactivation of multiple genes in skeletal muscle system. Thus, given the relative simplicity of generating gene specific depletion models using in vivo CRISPR/ Cas9 genome editing, we believe that this method will provide new avenues to further understand the mechanistic basis of many aspects of adult myogenesis.

In conclusion, we provide evidence that MICAL2 is a crucial modulator for myogenic differentiation in all the three muscle types. Translocation of MICAL2 to the nucleus is likely required to provide redox modification of nuclear actin and for its positive interaction with myogenic transcription factors. This regulatory switch is necessary in skeletal myogenic progenitors to stimulate myogenic differentiation and to elevate the expression of genes such as *Myogenin* and *MyH1*. However, the pathological nuclear localization indicates that MICAL2 is also necessary to proceed further in muscle regeneration probably boosting myogenic differentiation, as highlighted by our perturbation studies. Thus, MICAL2 is a novel regulator of skeletal myogenic differentiation and a possible therapeutic target for muscle disorders.

## Supplementary information

Supplementary Figure 1

Supplementary Figure 2

Supplementary Figure 3

Supplementary Figure 4

Supplementary Figure 5

Supplementary Figure 6

Supplementary figure legends

Supplementary tables

#5 beating CMs at d11

#6 beating CMs at d11

#GFP beating CMs at d11
