# Supplementary figures and images for: MICAL2 is essential for myogenic lineage commitment
Source: Cell Death Dis. 2020 Aug 18;11(8):654. doi: 10.1038/s41419-020-02886-z (PMC7434877; doi:10.1038/s41419-020-02886-z)

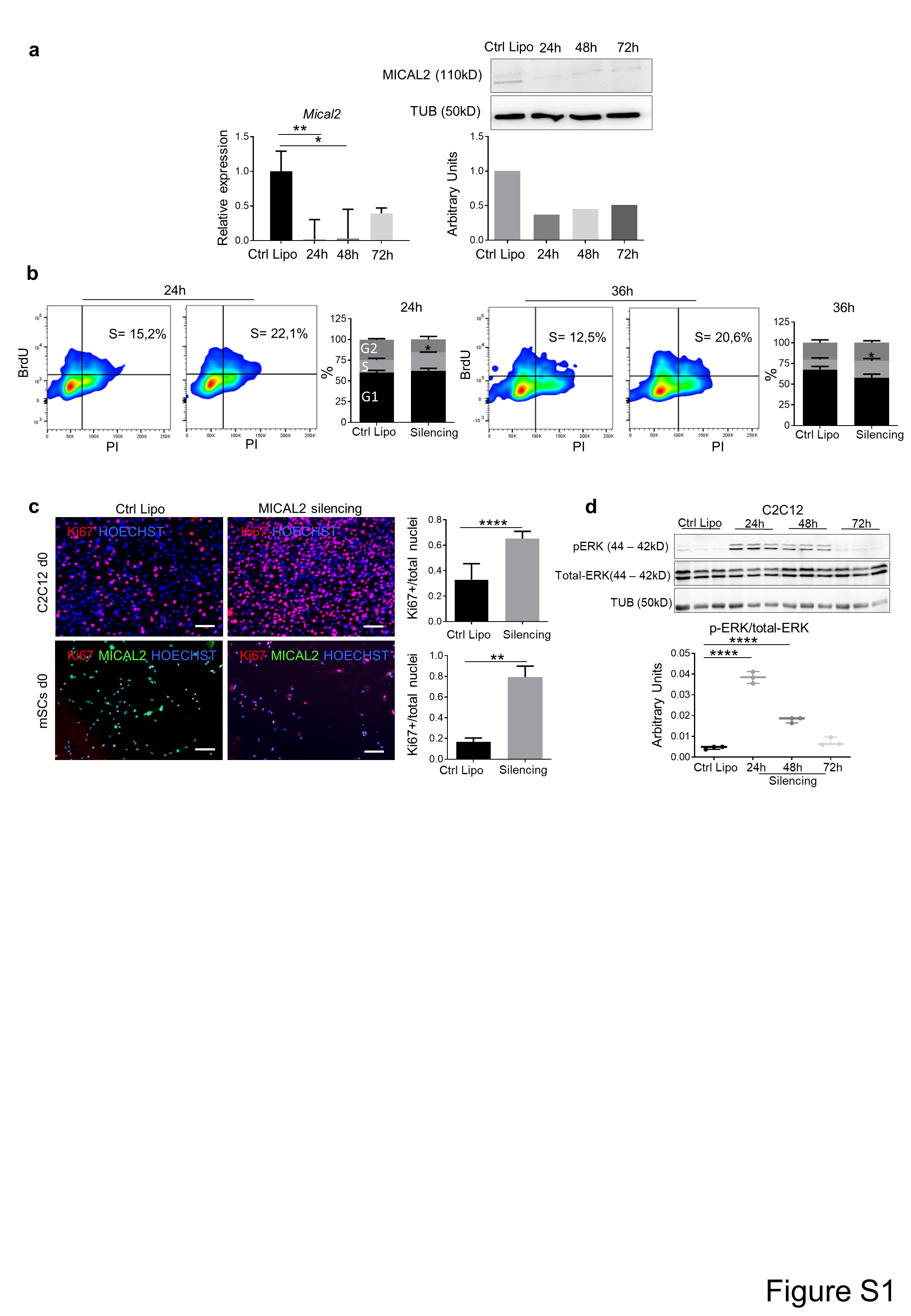

Supplement: Supplementary file 1 — Supplementary Figure 1 [file 41419_2020_2886_MOESM1_ESM.tif]

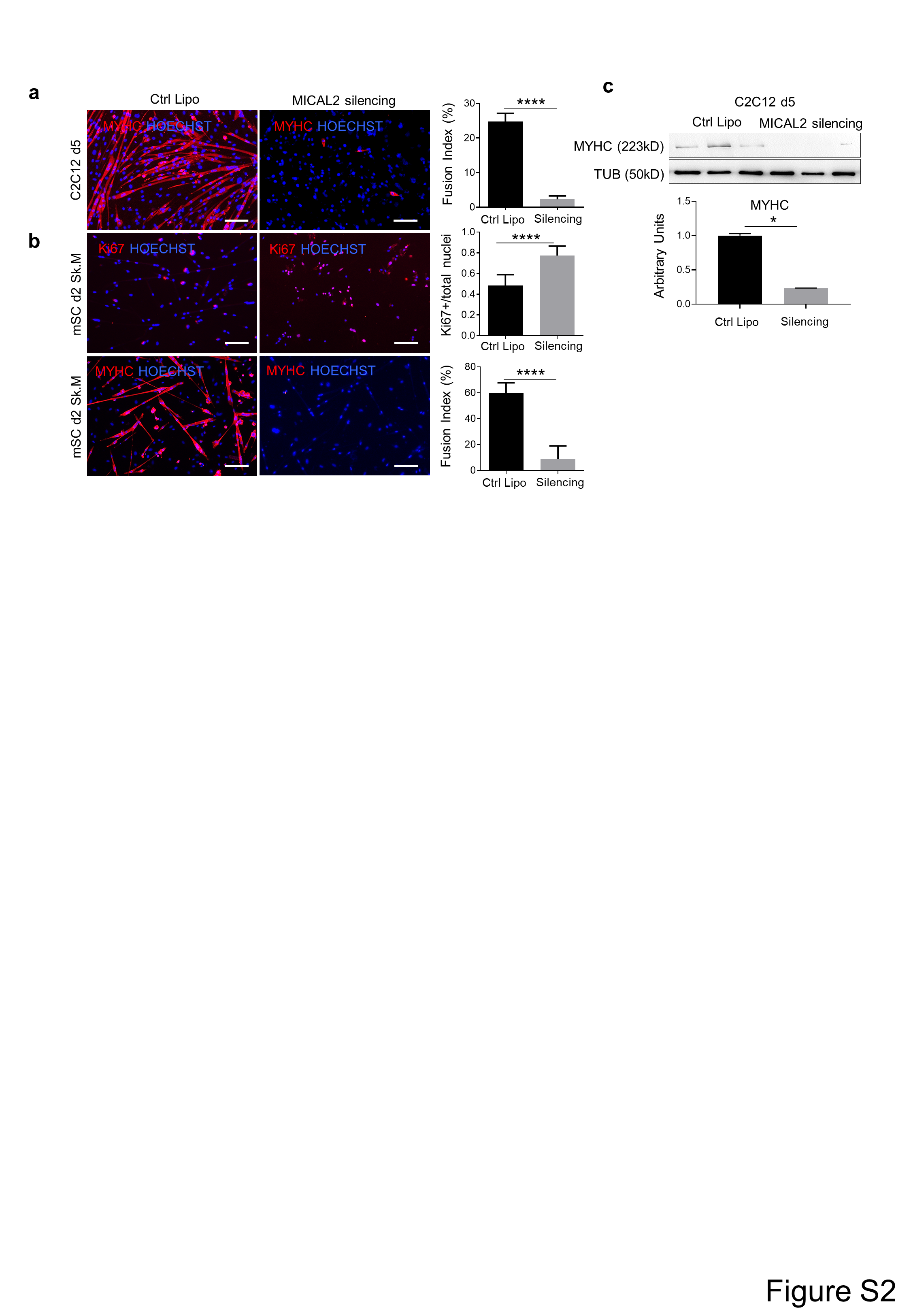

Supplement: Supplementary file 2 — Supplementary Figure 2 [file 41419_2020_2886_MOESM2_ESM.tif]

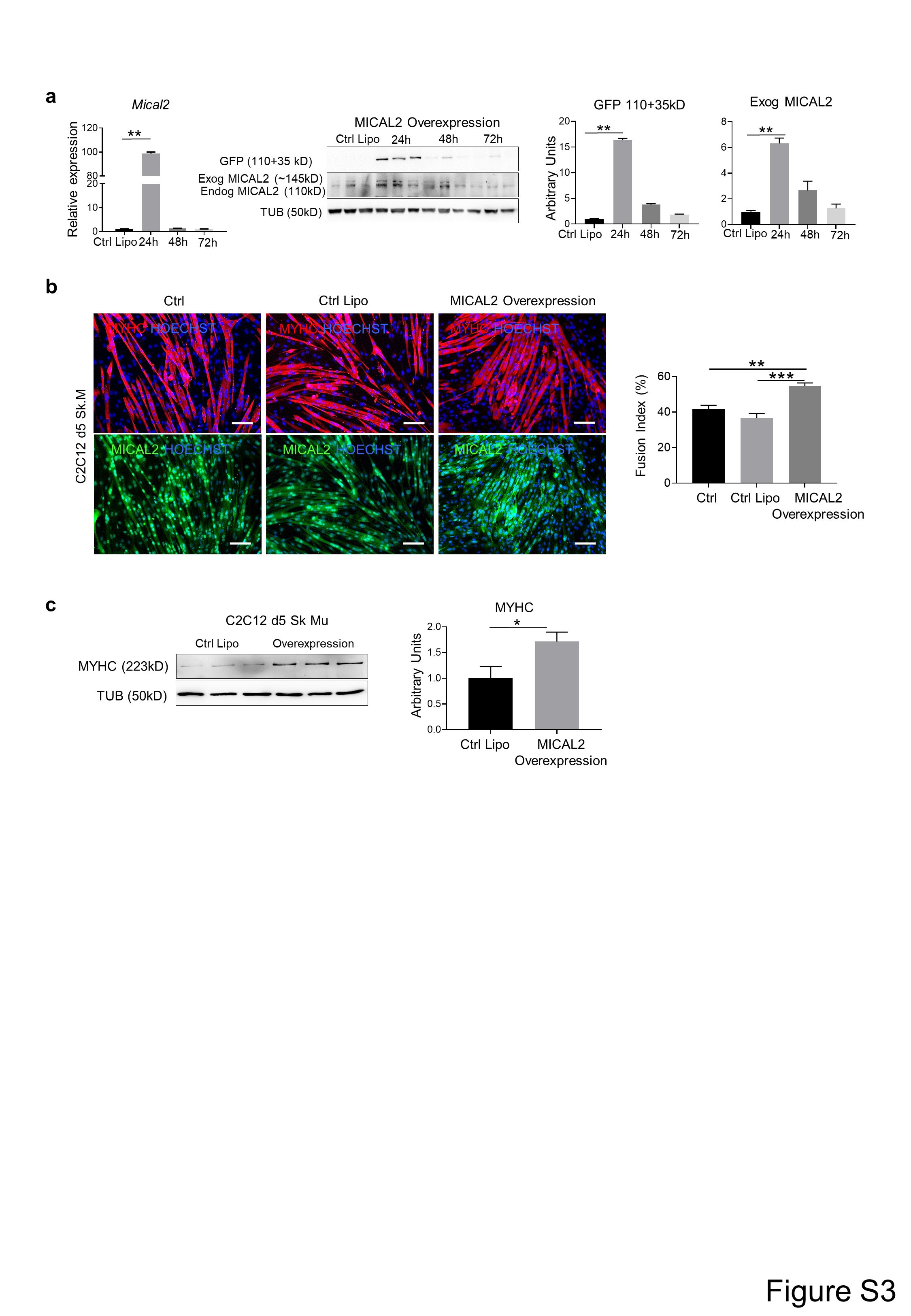

Supplement: Supplementary file 3 — Supplementary Figure 3 [file 41419_2020_2886_MOESM3_ESM.tif]

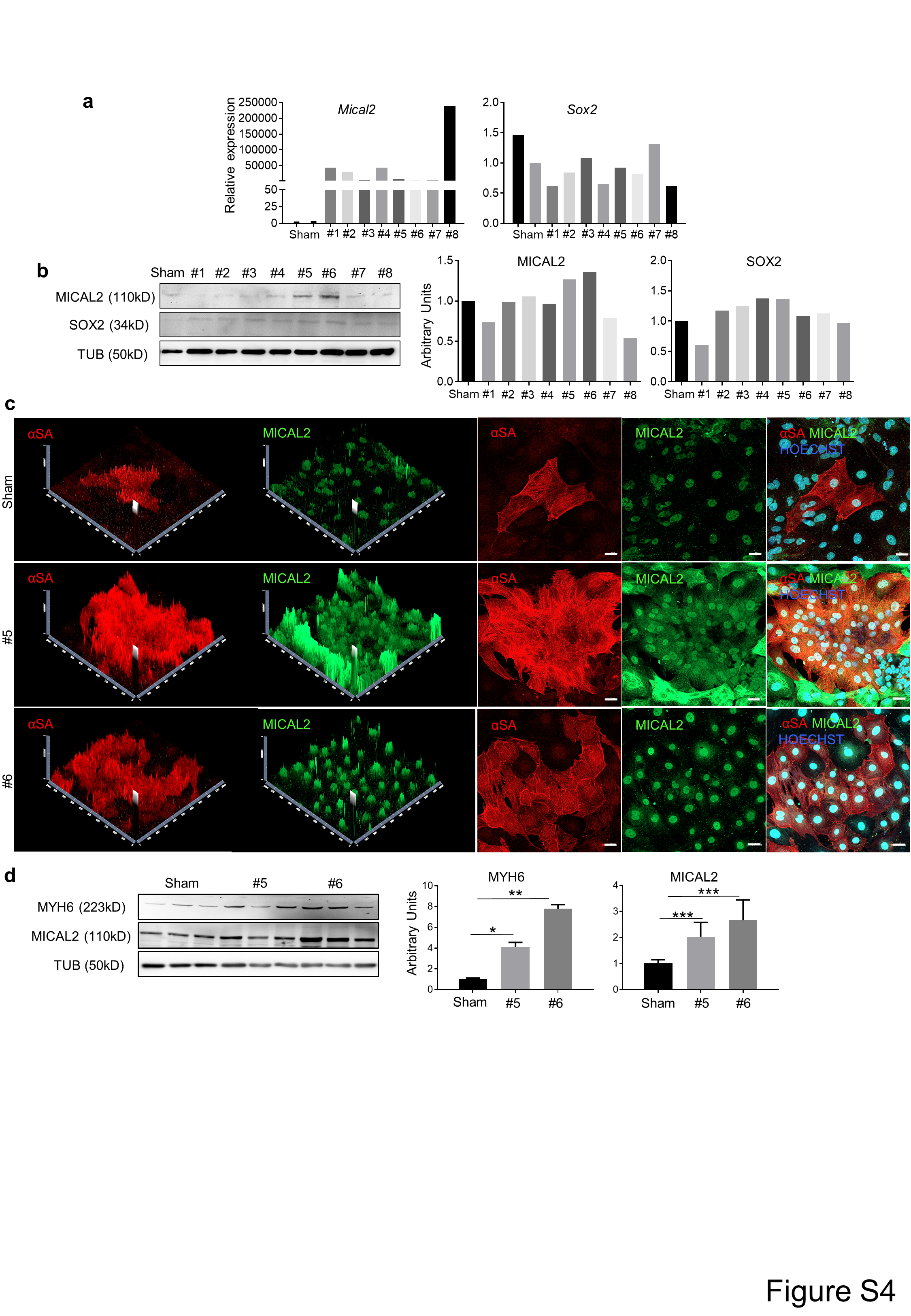

Supplement: Supplementary file 4 — Supplementary Figure 4 [file 41419_2020_2886_MOESM4_ESM.tif]

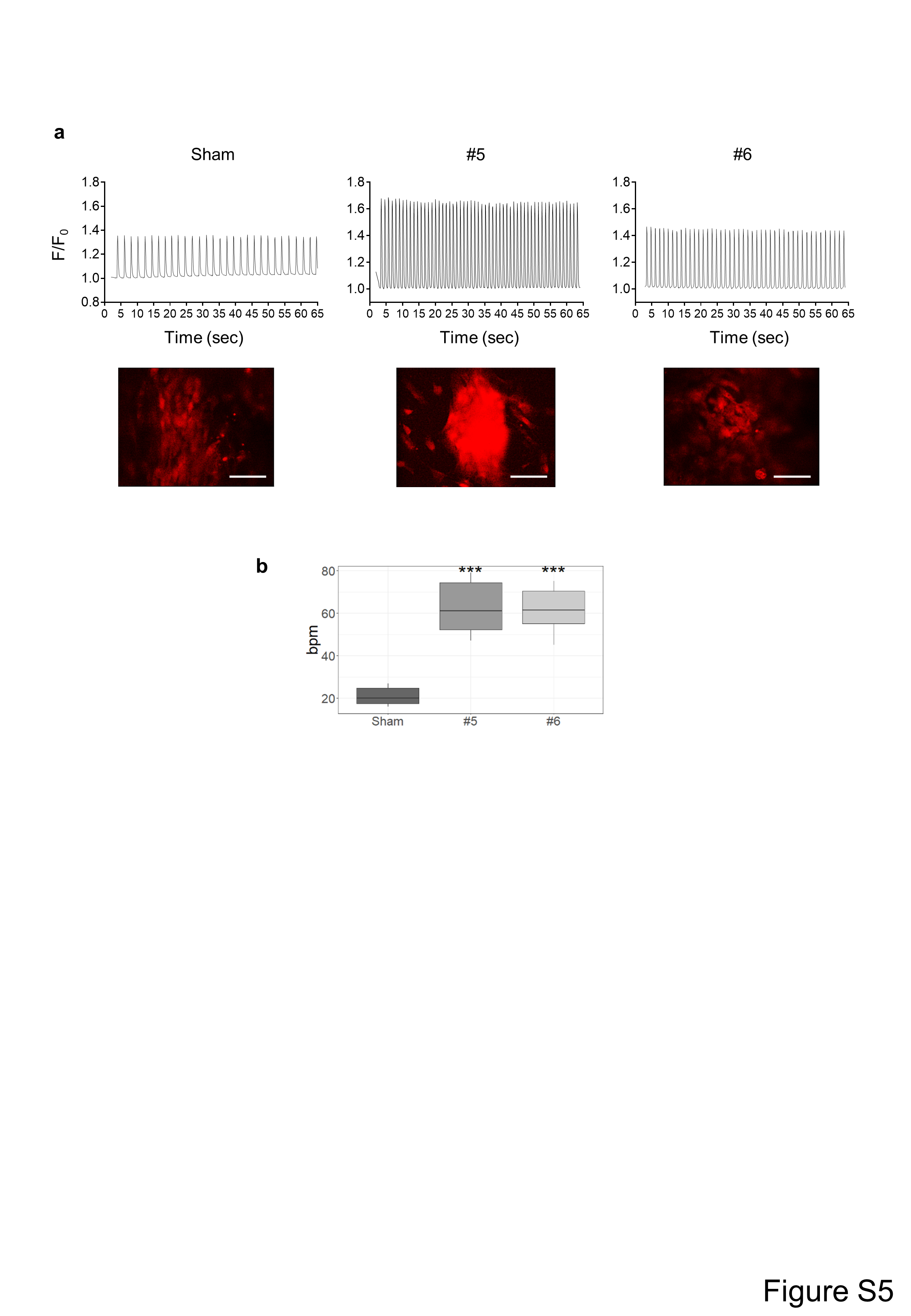

Supplement: Supplementary file 5 — Supplementary Figure 5 [file 41419_2020_2886_MOESM5_ESM.tif]

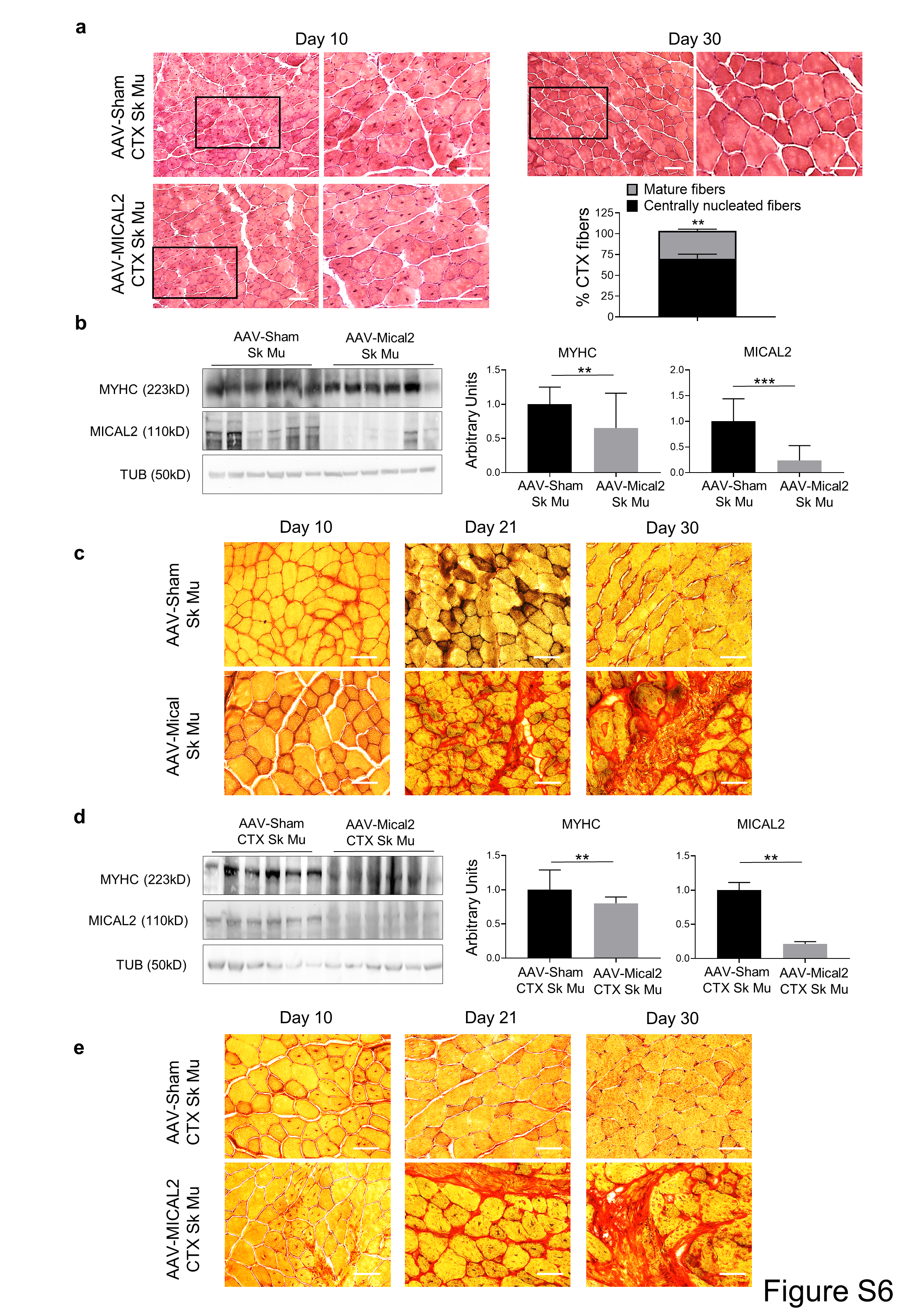

Supplement: Supplementary file 6 — Supplementary Figure 6 [file 41419_2020_2886_MOESM6_ESM.tif]
