# Supplementary material for: MICAL2 is essential for myogenic lineage commitment
Source: Cell Death Dis. 2020 Aug 18;11(8):654. doi: 10.1038/s41419-020-02886-z (PMC7434877; doi:10.1038/s41419-020-02886-z)
Supplement: Supplementary file 7 — Supplementary figure legends [file 41419_2020_2886_MOESM7_ESM.docx]

**Supplementary Figure 1: MICAL2 silencing enhances proliferation on C2C12 cells and mSCs. Related to Figure 1. a**) From left to right, qRT-PCR showing the relative expression and WB showing the protein content of *Mical2* indicating the efficiency of Mical2esiRNA silencing at 24h, 48h and 72h time points, compared to lipofectamine treated control cells (Ctrl Lipo). **b**) Flow cytometry analysis for BrdU incorporation in proliferating C2C12 cells at 24h and 36h after lipofectamine treatment for control cells (Ctrl Lipo) and Mical2esiRNA treatment for silenced cells (MICAL2silencing). Histograms indicate the % of cells in G1, S and G2 phases of cell cycle. N=3. **c**) In the upper panel, Ki67 (red) IF staining of lipofectamine treated control cells (Ctrl Lipo) and Mical2esiRNA treated cells (MICAL2silencing). Nuclei stained with HOECHST (blue). N=5. Lower panels show Ki67 (red) and MICAL2 (green) IF on proliferating mSCs Ctrl Lipo and MICAL2silencing. Nuclei stained with HOECHST (blue). N=5. Scale bars 200 μm. Quantification of proliferation is expressed as ratio between Ki67+ nuclei and total number of nuclei. **d**) WB on proliferating C2C12 cells after 24h, 48h and 72h of Mical2esiRNA compared to lipofectamine treated samples (Ctrl Lipo) for pERK and Total- ERK and relative ratio below. N=3. * = p<0,05; ** = p<0,01; **** = p<0,0001 by one-way ANOVA.

**Supplementary Figure 2: MICAL2 silencing impairs differentiation on C2C12 cells and mSCs. Related to Figure 1. a**) IF assay showing MyHC (red) staining of Ctrl Lipo and MICAL2 silencing C2C12 cells after five days of skeletal muscle differentiation. Nuclei stained with HOECHST (blue). N=5. FI quantification is expressed as percentage of the ratio between MyHC+ nuclei within myotubes and total number of nuclei. Scale bars 200 μm. **b**) Upper panels show IF for Ki67 (red) in Ctrl Lipo and MICAL2 mSCs after two days of skeletal muscle differentiation. Quantification of proliferation is expressed as ratio between Ki67+ nuclei and total number of nuclei. N=5. The lower panels show MyHC (red) IF staining in Ctrl Lipo and MICAL2 silencing mSCs after two days of skeletal muscle differentiation. Nuclei stained with HOECHST (blue). N=5. FI quantification is expressed as percentage of the ratio between MyHC+ nuclei within myotubes and total number of nuclei. Scale bars 200 μm. **c**) WB and relative quantification show MyHC protein in Ctrl Lipo and MICAL2 silencing C2C12 cells after five days of skeletal muscle differentiation. N=3. * = p<0,05; ** = p<0,01; *** = p<0,001; **** = p<0,0001 by two tailed t-test.

**Supplementary Figure 3: MICAL2 overexpression improves skeletal muscle differentiation in C2C12 cells. Related to Figure 1. a)** From left to right, qRT-PCR relative expression, WB and relative quantification of Mical2 gene and protein, respectively showing the efficiency of *Mical2* overexpressing plasmid at 24h, 48h and 72h time points, compared to Ctrl Lipo. In the WB, MICAL2-eGFP-fused protein and eGFP protein, both at ~140kD indicate exogenous MICAL2 in C2C12 cells at 24h, 48h and 72h time points, compared to Ctrl Lipo. N=3. **b**) From top to bottom, MYHC (red) and MICAL2 (green) IF staining of untreated control (Ctrl), Ctrl Lipo and MICAL2 overexpressing C2C12 cells after five days of skeletal muscle differentiation. Nuclei stained with HOECHST (blue). N=5. FI quantification is expressed as percentage of the ratio between MyHC+ nuclei within myotubes and total number of nuclei. Both Ctrl and Ctrl Lipo have been statistically compared to MICAL2 overexpressing C2C12 cells. Scale bars 200 μm. **c**) WB showing MYHC protein and relative quantification in Ctrl Lipo and MICAL2 overexpressing C2C12 cells after five days of skeletal muscle differentiation. N=3. * = p<0,05; ** = p<0,01; **** = p<0,0001 by one-way ANOVA test.

**Supplementary Figure 4: Overexpressing MICAL2 mESC lines reach more robust maturation towards cardiomyocytes-like cells. Related to Figure 3. a**) The qRT-PCT shows *Mical2* and *Sox2* relative expression in eight different pluripotent mESCs infected with pSRS11-EF1a-MmMICAL2 vector in comparison with a Sham control infected with pSRS1-SF-eGFP vector. **b**) WB for protein confirmation of MICAL2 overexpression and SOX2 in the same eight cell lines compared to the Sham control. Cell line #5 and #6 were chosen for further experiments. **c**) Fluorescence intensity histogram and IF showing α-SA (red) and MICAL2 (green) in Sham mESC line compared to line #5 and #6, after 11 days of cardiac differentiation. Nuclei stained with HOECHST (blue). N=3. Scale bars 20 μm. **d**) WB showing MYH6, MICAL2 protein and their relative quantification Sham versus #5 and #6 mESC line compared after 11 days of cardiac differentiation. N=3. * = p<0,05; ** = p<0,01; *** = p<0,001 by one-way ANOVA test.

**Supplementary Figure 5: Improved Ca^2+^ handling in cardiac differentiated MICAL2-mESC lines. Related to Figure 3. a**) Representative normalized traces of loaded Cal-590 Sham, #5, #6 mECS lines recorded for 65 seconds. Intracellular Ca^2+^ was analyzed in defined regions of interest that included comparable cluster of beating cells. Histograms show spontaneous changes in [Ca^2+^]_i_ expressed as F/F_0,_ where F is the fluorescence intensity normalized to the resting fluorescence (F_0_). Examples of Ca^2+^ fluorescence-intensity changes are reported below. Scale bars 200 μm. **b**) The box plots indicate beats per minutes (bpm) counted on 6 independent 1-minute-long movies of beating cardiomyocyte-like cells originated from Sham, #5 and #6 mESC lines. The movies are available in the supplemental material. N=6. *** = p<0,001; by one-way ANOVA test.

**Supplementary Figure 6: Fibrotic tissues accumulate in AAV-Mical2 treated skeletal muscles. Related to Figure 5 and 6. a**) H&E of AAV-Sham H11^Cas9^ and of AAV-Mical2 H11^Cas9^ TA muscles showing centrally nucleated fibers at 10 days from CTX injection that disappeared at day 30 only in AAV-Sham H11^Cas9^ muscles (a magnification of selected area of each image is reported on the right panels). The graph shows the percentage (%) of centrally nucleated fibers on the total number of fibers of 3 different mice at 10 day after CTX injury. Scale bars 50 μm. **b**) WB for MYHC and MICAL2 proteins on uninjured skeletal muscle of AAV-Sham H11^Cas9^ mice compared to AAV-Mical2 H11^Cas9^ mice. The relative quantification is on the right. N=6. **c**) Collagen deposition and fibrosis (red) in skeletal muscles are shown by Picro-Sirius red staining on left TA cross-sections of AAV-Sham H11^Cas9^ mice compared to AAV-Mical2 H11^Cas9^ mice, along time points of 10, 21 and 30 days. Scale bars 50 μm. **d**) WB for MYHC and MICAL2 proteins on CTX injured skeletal muscle of AAV-Sham H11^Cas9^ mice compared to AAV-Mical2 H11^Cas9^ mice. The relative quantification is on the right. N=6. **e**) Collagen deposition and fibrosis (red) in CTX injected skeletal muscles are shown by Picro-Sirius red staining on right TA cross-sections of AAV-Sham H11^Cas9^ mice compared to AAV-Mical2 H11^Cas9^ mice, along time points of 10, 21 and 30 days. Scale bars 50 μm. ** = p<0,01; by one-way ANOVA test.

**Supplemental information titles and legends**

**Supplementary Video 1. Beating frequencies of #5 cardiomyocyte-like cells.** Related to Figure S5B. Six independent beating clones of Mical2 overexpressing mESC line #5 were recorded at day 11 of cardiac differentiation for 60 seconds. These measurements were used to calculate the bpm showed in Figure S5B.

**Supplementary Video 2. Beating frequencies of #6 cardiomyocyte-like cells.** Related to Figure S5B. Six independent beating clones of Mical2 overexpressing mESC line #6 were recorded at day 11 of cardiac differentiation for 60 seconds. These measurements were used to calculate the bpm showed in Figure S5B.

**Supplementary Video 3. Beating frequencies of Sham cardiomyocyte-like cells.** Related to Figure S5B. Six independent beating clones of Mical2 overexpressing mESC Sham line were recorded at day 11 of cardiac differentiation for 60 seconds. These measurements were used to calculate the bpm showed in Figure S5B.
