# Supplementary material for: MICAL2 is essential for myogenic lineage commitment
Source: Cell Death Dis. 2020 Aug 18;11(8):654. doi: 10.1038/s41419-020-02886-z (PMC7434877; doi:10.1038/s41419-020-02886-z)
Supplement: Supplementary file 8 — Supplementary tables [file 41419_2020_2886_MOESM8_ESM.docx]

**Supplementary Table 1. List of primers**

| Gene | Primer orientation | Primer sequences 5’ > 3’ |
| --- | --- | --- |
| *α-Sma* | Forward | AGTCGCTGTCAGGAACCCTGAGACG |
|  | Reverse | ATCTTTTCCATGTCGTCCCAGTTG |
| *Anf* | Forward | ATTGACAGGATTGGAGCCCAGAGT |
|  | Reverse | TGACACACCACAAGGGCTTAGGAT |
| *Bax* | Forward | AAGCTGAGCGAGTGTCTCCG |
|  | Reverse | TGCCACCCGGAAGAAGACCT |
| *Bnp* | Forward | GTTTGGGCTGTAACGCACTGA |
|  | Reverse | GAAAGAGACCCAGGCAGAGTCA |
| *Brach* | Forward | TGCTGCCTGTGAGTCATA |
|  | Reverse | ACAAGAGGCTGTAGAACATG |
| *Calponin* | Forward | ACATCATTGGACTGCAGATG |
|  | Reverse | CAAAGATCTGCCGCTTGGTG |
| *Cas9* | Forward | TCATCCGCCGCTCGATGAAGCTC |
|  | Reverse | AAACAGCAGATTCGCCTGGA |
| *Casp3* | Forward | GGAGTCTGACTGGAAAGCCGAA |
|  | Reverse | CTTCTGGCAAGCCATCTCCTCA |
| *Ccl2* | Forward | TTAAAAACCTGGATCGGAACCAA |
|  | Reverse | GCATTAGCTTCAGATTTACGGGT |
| *CD45* | Forward | ACCACAAGTTTACTAACGCAAGT |
|  | Reverse | TTTGAGGGGGATTCCAGGTAAT |
| *Gapdh* | Forward | TGGTGAAGGTCGGTGTGAAC |
|  | Reverse | GCTCCTGGAAGATGGTGATGG |
| *Hprt* | Forward | TGGATACAGGCCAGACTTTGTT |
|  | Reverse | CAGATTCAACTTGCGCTCATC |
| *Icam1* | Forward | TGGAGACGCAGAGGACCTTA |
|  | Reverse | CGCTCAGAAGAACCACCTTC |
| *Il10* | Forward | ATCGATTTCTCCCCTGTGAA |
|  | Reverse | TGGCCTTGTAGACACCTTGG |
| *Inf-γ* | Forward | GCGTCATTGAATCACACCTG |
|  | Reverse | CTGGACCTGTGGGTTGTTG |
| *Mef2c* | Forward | ATGAGCGTAACAGACAGG |
|  | Reverse | TTATCCATGTCAGTGCTGG |
| *Mical2* | Forward | AAAGAGAGGAGGAAGGAACATGG |
|  | Reverse | GAACTGGAAGGCTGAAACGTA |
| *MixL1* | Forward | ACCACCAGGCCTGACAACCT |
|  | Reverse | TGGGTGCACACCATACCACA |
| *MyHC* | Forward | TTCATTAGTTTCCCAGCTCTCC |
|  | Reverse | AGGCACTCTTGGCCTTTATC |
| *Myh6* | Forward | ATCTCTGACAACGCCTATC |
|  | Reverse | GATAGGCGTTGTCAGAGAT |
| *MyoD* | Forward | GAGCAAAGTGAATGAGGCCTT |
|  | Reverse | CACTGTAGTAGGCGGTGTCGT |
| *Myogenin* | Forward | ATGGAGCTGTATGAGACATCCCC |
|  | Reverse | CGACACAGACTTCCTCTTACAC |
| *Nanog* | Forward | GAGTGTGGGTCTTCCTGGTC |
|  | Reverse | GAGGCAGGTCTTCAGAGGAA |
| *Oct4* | Forward | CCAGGCAGGAGCACGAGTGG |
|  | Reverse | CCACGTCGGCCTGGGTGTAC |
| *Pax3* | Forward | TGCGTCGTCTCTAAGATCCTGTGCAG |
|  | Reverse | CAGCTCTGCGAAGGTGGT |
| *Pax7* | Forward | CCCTCAGTGAGTTCGATTAGCC |
|  | Reverse | GGTCGGGTTCTGATTCCACA |
| *Sm22* | Forward | TCCAGTCCACAAACGACCAAGC |
|  | Reverse | GAATTGAGCCACCTGTTCCATCTG |
| *Sm-Myhc* | Forward | TCAACGCCAACCGCAGGAAGCTG |
|  | Reverse | TGCTAAGCAGTCTGCTGGGCT |
| *Sox2* | Forward | CTGTTTTTTCATCCCAATTGCA |
|  | Reverse | CGGAGATCTGGCGGAGAATA |
| *Tbp* | Forward | CAAACCCAGAATTGTTCTCCTT |
|  | Reverse | ATGTGGTCTTCCTGAATCCCT |
| *Tgf-β1* | Forward | TTGCTTCAGCTCCACAGAGA |
|  | Reverse | TGGTTGTAGAGGGCAAGGAC |
| *Tnf-α* | Forward | CCCTCACACTCAGATCATCTTCT |
|  | Reverse | GCTACGACGTGGGCTACAG |
| *Tnnt3* | Forward | GCGTTCTGAGGACTCGTTG |
|  | Reverse | CGTGAAGCTGTCGGCATAAG |

**Supplementary Table 2. List of primary antibodies for WB and IF assays**

| Primary antibody | WB dilution | IF dilution |
| --- | --- | --- |
| rabbit anti-α-SMA | 1:800 | - |
| mouse anti-α-SMA Cy3 conjugated | - | 1:200 |
| mouse anti-α-SARCOMERIC ACTININ | - | 1:100 |
| mouse anti-α-TUBULIN | 1:1000 | - |
| rabbit anti-GFP | 1:1000 | - |
| Hoechst | - | 1:10000 |
| mouse anti-Ki67 | - | 1:300 |
| rabbit anti-LAMININ | - | 1:300 |
| mouse anti-MF20 | 1:3 | 1:20 |
| rabbit anti-MICAL2 (OriGene) | 1:500 | - |
| rabbit anti-MICAL2 (SantaCruz) | - | 1:50 |
| mouse anti-MyH6 | 1:500 | 1:100 |
| mouse anti-p-ERK | 1:300 | - |
| goat anti-SOX2 | 1:500 | 1:50 |
| rabbit anti-Total-ERK | 1:300 | - |
